# Comparative prebiotic potential of galacto- and fructo-oligosaccharides, native inulin, and acacia gum in Kenyan infant gut microbiota during iron supplementation

**DOI:** 10.1093/ismeco/ycae033

**Published:** 2024-03-11

**Authors:** Paula Momo Cabrera, Carole Rachmühl, Muriel Derrien, Raphaëlle Bourdet-Sicard, Christophe Lacroix, Annelies Geirnaert

**Affiliations:** Laboratory of Food Biotechnology, Institute of Food, Nutrition and Health, ETH Zurich, 8092 Zurich, Switzerland; Laboratory of Food Biotechnology, Institute of Food, Nutrition and Health, ETH Zurich, 8092 Zurich, Switzerland; Danone Global Research & Innovation Center, 91190 Gif sur Yvette, France; Present address: Department of Microbiology and Immunology, Laboratory of Molecular Bacteriology, Rega Institute KU, 3000 Leuven, Belgium; Danone Global Research & Innovation Center, 91190 Gif sur Yvette, France; Laboratory of Food Biotechnology, Institute of Food, Nutrition and Health, ETH Zurich, 8092 Zurich, Switzerland; Laboratory of Food Biotechnology, Institute of Food, Nutrition and Health, ETH Zurich, 8092 Zurich, Switzerland

**Keywords:** ex vivo, gut microbiota model, dietary fibers, short-chain fatty acids, bifidogenic, non-Western infant microbiome, individual microbiota response, iron deficiency anemia, pre-clinical study, dose-response

## Abstract

Iron fortification to prevent anemia in African infants increases colonic iron levels, favoring the growth of enteropathogens. The use of prebiotics may be an effective strategy to reduce these detrimental effects. Using the African infant PolyFermS gut model, we compared the effect of the prebiotics short-chain galacto- with long-chain fructo-oligosaccharides (scGOS/lcFOS) and native inulin, and the emerging prebiotic acacia gum, a branched-polysaccharide–protein complex consisting of arabinose and galactose, during iron supplementation on four Kenyan infant gut microbiota. Iron supplementation did not alter the microbiota but promoted *Clostridioides difficile* in one microbiota. The prebiotic effect of scGOS/lcFOS and inulin was confirmed during iron supplementation in all investigated Kenyan infant gut microbiota, leading to higher abundance of bifidobacteria, increased production of acetate, propionate, and butyrate, and a significant shift in microbiota composition compared to non-supplemented microbiota. The abundance of the pathogens *Clostridium difficile* and *Clostridium perfringens* was also inhibited upon addition of the prebiotic fibers. Acacia gum had no effect on any of the microbiota. In conclusion, scGOS/lcFOS and inulin, but not acacia gum, showed a donor-independent strong prebiotic potential in Kenyan infant gut microbiota. This study demonstrates the relevance of comparing fibers *in vitro* prior to clinical studies.

## Introduction

Iron-deficiency anemia (IDA) is a severe condition that can lead to impaired cognitive and motor development [1, 2]. An estimated 20%–25% of children worldwide suffer from IDA, with the highest prevalence reported in low- and middle-income countries of South Asia and Africa [3]. Kenya has a particularly high IDA prevalence, with reported estimates between 62% and 73% among infants aged 8–12 months [4, 5]. Supplementing complementary food with iron-containing micronutrient powders is a common strategy to reduce IDA in African infants during weaning [6, 7]. However, such iron fortification sharply increases the colonic iron levels, as typical absorption in the small intestine is 10%–20% [8]. Unabsorbed iron remains available for gut microbes and may potentially affect the gut microbiome homeostasis [9, 10].

Indeed, studies in Kenyan infants aged 6 months and 6.5–9.5 months reported adverse effects with dietary iron fortification (12.5 and 5 mg iron/day) on the gut microbiota, with decreased abundance of infant health-associated bifidobacteria and lactobacilli concomitant with increased *Enterobacteriaceae* and pathogenic *Escherichia coli* [11–13]. Similarly, supplementation with 12.5 mg iron/day in the form of micronutrient powder resulted in increasing abundance of *Escherichia–Shigella* and decreased *Bifidobacterium* in 6-month-old Pakistani infants [14]. Although the exact mechanism of the observed iron-induced dysbiosis and adverse gut events are not fully characterized, strategies to improve the safety of iron fortification are currently being investigated.

Iron is essential for fundamental processes in both mammalian and microbial cells, and its homeostasis is tightly regulated [15, 16]. For example, iron is a cofactor in iron-dependent enzymes involved in the butyrate production pathways and its supplementation promoted butyrate gut levels in rats [17, 18] and butyrate production by *Roseburia intestinalis in vitro*, while pronounced iron deficiency inhibited *in vitro* butyrate production by the gut microbiota of children [19]. Most enteropathogenic bacteria, such as *Salmonella*, *Shigella*, and pathogenic *E. coli*, rely on iron acquisition for gut colonization and for the expression of virulence genes [20]. Therefore, excess iron in the gut lumen may stimulate the colonization by and virulence of various pathogenic bacteria [8, 21]. In contrast, beneficial commensal gut bacteria such as lactobacilli were shown to require iron only under particular environmental conditions [22], and bifidobacteria isolated from Kenyan infant feces efficiently bound iron *via* siderophores, potentially limiting the iron availability to pathogens [23]. Thus, higher colonic iron levels resulting from fortification may shift the colonic microbiota equilibrium to favor the growth and virulence of pathogenic strains over healthy barrier-protecting strains in infants living in rural areas of low- and middle-income countries.

Applying prebiotics is a potent strategy to stimulate the growth and colonization of specific bacteria in the gut to confer a health benefit [24], and to promote production of short-chain fatty acids (SCFAs). Such acids decrease colonic pH, which can lead to inhibition of enteropathogens [25] and improved iron absorption [26]. Adding prebiotic galacto-oligosaccharides (GOS) to a micronutrient powder containing a lower dose of highly bioavailable iron (5 mg/day) partially prevented enteropathogen stimulation by iron supplementation in Kenyan infants; treatment with GOS resulted in higher abundances of *Bifidobacteriaceae* and *Lactobacillaceae* and lower abundances of pathogenic virulence and toxin genes [4, 12]. The prebiotic capacity of short-chain GOS combined with long-chain fructo-oligosaccharides (FOS) (scGOS/lcFOS) [27, 28] and of inulin [29] was demonstrated in trials involving Western infants; however, this same effect has not yet been investigated in rural African infant cohorts, which have different gut microbial compositions including different enteropathogens [12, 30]. Compared to GOS, acacia gum, an arabinogalactan–protein complex, is a more affordable emerging prebiotic that can be fermented by human adult microbiota [31–33] and has a demonstrated bifidogenic effect in human adults [34, 35]. Therefore, it is also a potent prebiotic candidate for its ability to promote barrier-protective bacterial taxa and its functions during iron fortification in the African infant gut microbiota.

*In vitro* gut microbiota models are effective tools for studying the human gut microbiota function and composition in a controlled environment independent of the host [36]. We recently adapted the *in vitro* continuous gut fermentation model PolyFermS to mimic conditions of infant gut microbiota from rural Kenya through protected transport of fresh fecal samples and adaptation of *in vitro* cultivation medium and fermentation conditions to the Kenyan infant diet [37, 38].

Therefore, the aim of this study was to investigate the effect of prebiotics (scGOS + lcFOS, inulin) and the emerging prebiotic acacia gum during iron supplementation at a dose recommended by the World Health Organization (12.5 mg iron/day to reduce IDA in 6- to 23-month-old infants) on the gut microbiota of African infants at weaning age using the African infant PolyFermS model. We hypothesized that the selected fibers promote growth and metabolism of health-associated infant gut bacteria and inhibit the growth of opportunistic enteropathogens.

## Materials and methods

### Fecal sample collection, transport, and processing

Fresh fecal samples from four partially breastfed Kenyan infants donors (D1, D2, D3, and D4) aged between 4.8 and 7.9 months living in rural Kenya (Msambweni County) were collected and processed as previously described [37] and were part of the African infant PolyFermS model setup study [38]. None of the infants received antibiotics or dietary supplements prior to sample donation; detailed fecal donor information is presented in supplemental data. The fecal samples were collected in context of the registered clinical trial on clinicaltrials.gov (NCT03866837) and from participants prior to intervention. The Ethics Commission of ETH Zürich, Switzerland (EK 2019-N-04) and the Kenya Medical Research Institute (KEMRI) Scientific and Ethics Review Unit (SERU) (KEMRI/RES/7/3/1 no. 656) reviewed and approved this study. Informed consent was obtained from the parents or the legal guardians of the infants.

### PolyFermS *in vitro* colonic fermentation experiments

Four independent PolyFermS systems were inoculated with immobilized fecal microbiota from the four donors and operated to model the Kenyan infant gut microbiota as described before [38]. In short, each infant’s fecal microbiota was immobilized into 1–2 mm diameter gellan–xanthan gum gel beads under anaerobic conditions. The fecal gel beads were used to inoculate (30% v/v) the inoculum reactor (IR) of a Multifors Bioreactor System (D2 and D3, Multifors, Infors, Bottmingen, Switzerland) or DASbox® Mini Bioreactor System (D1 and D4, Eppendorf, Juelich, Germany). The bioreactors contained a nutritive medium that mimicked chyme entering the colon of a Kenyan infant aged 4–9 months that fed daily on 0.5 L human milk and 300 g maize porridge, complemented with fruits and vegetables, which was prepared as previously described [38]. The medium was composed of (g/L of dH2O): zein (corn protein, 0.3), gluten hydrolysate from corn (0.3), corn starch (0.3), xylan (oat spelt, 0.4), arabinogalactan (larch wood, 2.2), D-lactose (3.2), casein hydrolysate (0.3), whey protein hydrolysate (4.1), peptone from casein (0.5), bactotryptone (0.5), mucin (4.0), yeast extract (standard nucleotide, 2.5), L-cysteine HCl (0.8), 0.05 bile salts, KH2PO4 (0.5), NaHCO3 (1.5), NaCl (4.5), KCl (4.5), MgSO4 (1.3), CaCl_2_·2H_2_O (0.1), hemin (0.01), Tween 80 (1.0), and vitamin solution (0.5 ml/L, composition previously described [38]). After two consecutive batch fermentations (pH 5.8, mixing at 180 rpm, and 37°C), continuous fermentation was started by continuously supplying fresh nutritive medium at a flow rate of 25 ml/h and removing the same volume of fermented medium, with a working volume of 200 ml and a mean retention time of 8 h [38]. Anaerobiosis was maintained by continuously supplying CO_2_ to the headspace, while redox potential was continuously monitored by EasyFerm® Plus-ORP Arc 120 sensors (Hamilton, Bonaduz, Switzerland). After a minimum stabilization period of 15 days, which was defined by stable fermentation metabolite production (<10% day-to-day variation), the IR effluent was used to continuously inoculate (5% (v/v)) seven parallel second-stage test reactors (TR) that were operated at identical conditions and connected to IR *via* a peristaltic pump. All TRs were additionally continuously supplied with 95% (v/v) nutritive medium.

### Experimental design for investigating prebiotic effects

**Figure 1:**
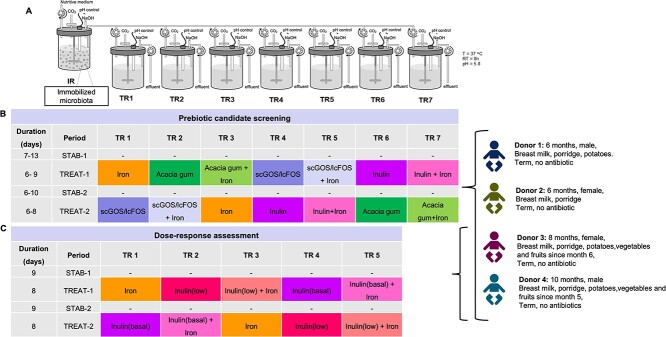
Overview of experimental set-up of PolyFermS *in vitro* model. (A) IR with immobilized fecal Kenyan infant microbiota and parallel second-stage test reactors (TR1-TR7). (B) Assessment of the effect of prebiotic screening of acacia gum, inulin, and scGOS/lcFOS in combination with iron supplementation for both treatment periods. The supplemented dose for all fibers was mimicking 5 g/d and for iron 12.5 mg/d. (C) Dose–response assessment for inulin with low dose 2.5 g/d versus basal dose 5 g/d. STAB: stabilization period; TREAT: treatment period.

The prebiotic potential of three different fibers: acacia gum (FIBREGUM LI, Nexira, France), short-chain GOS (Vivinal GOS powder, Friesland-Campina, The Netherlands) combined with long-chain FOS (Orafti HP, Beneo, Belgium) (scGOS/lcFOS at a 9:1 ratio), and native inulin (Orafti ST, Beneo, Belgium), alone and in combination with iron, were tested. Seven treatments were tested in parallel in the TRs: iron, acacia gum, acacia gum + iron, inulin, inulin + iron, scGOS/lcFOS, and scGOS/lcFOS + iron, all repeated during two experimental phases (Fig. 1). Each phase consisted of a stabilization period (6–13 days) followed by a treatment period (6–9 days). The fibers were supplemented to mimic a daily consumption dose of 5 g /infant. Considering a retention time of 8 h [39] and an infant colon capacity of 300 ml [40], the daily dosage was calculated to be 3.3 g/day/reactor or 5.5 g/l of medium feeding the reactors. The purity of the fiber powders (acacia gum: 100%, scGOS:lcFOS: 70% and inulin: 95%) was taken into account when calculating medium supplementation rates (Table S2). The iron supplementation mimicked oral supplementation of 12.5 mg elemental iron per day, of which on average 20% is absorbed in the duodenum and 80% enters the colon. The amount of iron in the medium was calculated with the retention time and infant colon volume, as explained above. Therefore, filter-sterilized (0.2 μm) iron solution (FeSO_4_^*^ 7 H_2_O, Sigma-Aldrich) was added to nutritive medium, resulting in 62.25 mg FeSO_4_^*^ 7 H_2_O/L. After testing all three fibers in the four different donor microbiota, a lower dose of inulin was also tested (2.5 g/day) and compared to the basal dose (5 g/day) provided to the microbiota in D3 and D4 reactors.

Each stabilization and treatment period lasted for 6 to 13 days, which was when the metabolic pseudo-steady state, which was monitored by the day-to-day variation of fermentation metabolites, was reached. Variations in day-to-day metabolite production in PolyFermS models are expected to be lower than 10%. This criterium was used to define functional microbial stability and the start or end of a treatment period. Between each treatment period, TRs were disconnected from IR, washed, sterilized and re-connected to the IR to avoid any effects of the previous applied treatment and re-establish the bacterial composition during the consecutive stabilization period.

Effluent samples from all PolyFermS reactors were taken daily. Samples were centrifuged (14 000 x *g* for 10 min at 4°C), and the supernatant was immediately processed or stored at −20°C, while the bacterial pellet was stored at −80°C until further analysis.

### Microbiota metabolite analysis

Concentrations of SCFA (acetate, butyrate, propionate), intermediate fermentation metabolites (formate, succinate, lactate) and branched-chain fatty acids (isobutyrate, isovalerate, valerate) were determined in PolyFermS effluent supernatant and fecal samples with a LaChrom high-performance liquid chromatography-system (Merck-Hitachi, Tokyo, Japan) was used with a Security Guard Cartridge Carbo-H (4 × 3.0 mm; Phenomenex Inc., Torrance, CA, United States) connected to a Rezex ROA-organic acid H+ column (300 x 7.8 mm; Phenomenex Inc., Torrance, CA, USA) and an Accela RI detector (Thermo Fisher Scientific Inc., Waltham, MA, USA). Sample volumes of 20 μl were analyzed with a mobile phase of 10 mM H_2_SO_4_ at a flow rate of 0.6 ml/min and a column temperature of 40°C. The data were processed using EZChrom software (Agilent, Santa Clara, CA, USA).

### Microbiota composition analysis

The DNA from feces and effluent pellets was extracted using the Fast DNA TM Spin Kit for Soil (MP Biomedicals, Illkirch-Graffenstaden, France) following the supplier’s protocol. The DNA concentration was measured with Nanodrop (ND-1000, Witec AG, Sursee, Switzerland), and samples were diluted to 20 ng DNA/μl using DES water (Fast DNA TM Spin Kit, MP Biomedicals) and stored at −20°C until further processing.

DNA from feces and IR effluent (days 21–23) was screened for presence of pathogens *Clostridioides difficile, Clostridium perfringens,* Enteropathogenic *E. coli* (EPEC)*, Salmonella* spp., enterotoxigenic *E. coli, Staphylococcus aureus,* enterohemorrhagic *E. coli* and *Campylobacter* by PCR, using primers described in Table S3.

Selected bacterial targets were further quantified with qPCR in feces and PolyFermS effluent from the last three days of stabilization and treatment periods with primers listed in in Table S3. Bacterial communities in the fecal and PolyFermS samples were analyzed with V4 16S rRNA gene amplicon sequencing with an Illumina MiSeq platform (Genetic Diversity Centre, ETH Zurich, Switzerland) and described in supplementary methods.

### Statistical analysis

Statistical analyses were performed in R version 4.2.0 with RStudio 2022.2 and GraphPad 9.2.0 and details are given in supplementary methods. Each dataset was first tested for normality using the Shapiro–Wilk test. Because data were not normally distributed, the nonparametric Wilcoxon rank sum test with false-discovery rate correction was used. *P* values <0.05 were considered statistically significant.

## Results

### Individual donor fecal microbiota profiles were reproduced and maintained *in vitro*

To assess the effect of the selected fiber mixtures on the target gut microbiota, we used the validated *in vitro* continuous PolyFermS fermentation model for African infant gut microbiota [38]. We performed four independent PolyFermS experiments with IRs containing immobilized Kenyan infant fecal microbiota. Basal data for untreated IR were previously described [38].

**Figure 2:**
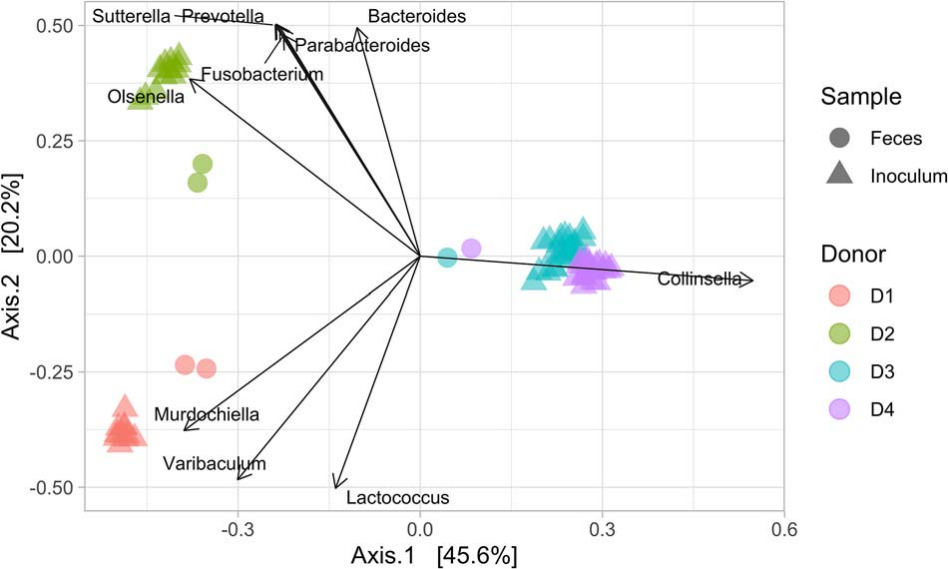
Differences in four donor *in vitro* baseline microbiota. Beta diversity represented as PCoA of binary Jaccard distance metrices comparing the 16S rRNA gene composition of feces and *in vitro* PolyFermS IR (inoculum) microbiota samples of all four donors (D1, D2, D3, and D4). Vectors indicate the top 10 genera that determine the binary Jaccard community structure. Data were derived from [38].

The inter-individual differences in composition between the fecal microbiota were maintained in the IR for all four donors, as indicated by different sample clusters in the binary Jaccard principal coordinates analysis (PCoA) plot (Fig. 2), with donor fecal samples that clustered with the corresponding *in vitro* model microbiota. As an example, the inter-donor distance between D1 and D2 fecal microbiota was 0.7 ± 0.004, while for *in vitro* microbiota it was 0.95 ± 0.01 (Fig. S1A). The D3 and D4 microbiota clustered closer together, which indicates a similar taxa composition. *In vitro* microbiota of these two donors were characterized by *Collinsella*, *Lactococcus*, *Veillonella*, and *Bifidobacterium*, but also *Bacteroides* in the case of D3 (Fig. S2). In contrast, the *in vitro* D1 microbiota was dominated (relative abundance) by *Streptococcus*, *Bifidobacterium, Veillonella,* and *Lactococcus*; and D2 *in vitro* microbiota by *Bacteroides*, *Olsenella*, *Megasphaera*, *Lactococcus*, *Hungatella*, and *Prevotella* (Fig. S2). The alpha diversity measured as observed amplicon sequencing variants (ASV) richness and Shannon index (H′) was lowest for D1 and D3 microbiota (observed ASVs: 39 ± 3 and 42 ± 4; H′: 2.3 ± 0.1 and 2.5 ± 0.1) compared to the D2 and D4 *in vitro* microbiota (observed ASVs: 53 ± 2 and 50 ± 3; H′: 2.7 ± 0.1 and 2.6 ± 0.1) (Fig. S1B).

Different opportunistic enteropathogens were detected at different concentrations in the *in vitro* microbiota. *C. perfringens* was detected in D1, D3, and D4 *in vitro* baseline microbiota at concentrations of 8.68 ± 0.06; 7.33 ± 0.24; and 3.17 ± 3.22 log(bacteria/ml) respectively, whereas *Clostridioides difficile* was detected in D1, D2, and D4 *in vitro* microbiota at baseline concentrations of 3.30 ± 0.20; 7.98 ± 0.47; and 8.27 ± 0.38 log(cells/ml), respectively. EPEC was only detected in baseline microbiota D3 (6.64 ± 0.07 log(cells/ml)) and D4 (5.70 ± 0.35 log(cells/ml)).

The fermentation metabolite profile for D1, D3, and D4 microbiota was characterized as propiogenic (propionate as main end metabolite after acetate) and for D2 microbiota as butyrogenic (butyrate as main end metabolite after acetate) (Fig. S1C). Over the course of the different experimental periods, the four different IR microbiota had stable fermentation metabolite production and stable abundant genera composition (Figs S2 and S3). Overall, our study consisted of *in vitro* microbiota that differed on alpha-diversity, pathogens load and SCFA and thus allows us to test the prebiotic effects of the three fibers and possible donor specificity.

### Consistent bifidogenic and SCFA-promoting effects of scGOS/lcFOS and inulin supplementation across all donors

**Figure 3:**
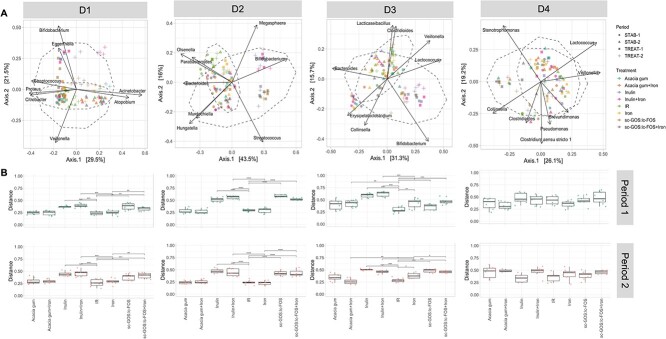
Overall microbiota composition response. (A) PCoA of weighted Jaccard distance metric assessing donor-dependent treatment effect upon supplementation of iron and of acacia gum, scGOS/lcFOS and inulin alone and in combination with iron in microbial composition, and main taxonomic drivers of intra-donor compositional differences in D1, D2, D3, and D4. (B) Weighted Jaccard distance metric assessing the intra-treatment induced microbiota shifts for D1, D2, D3, and D4 in period 1 (above) and period 2 (below). The values indicate the distance between the community at the end of stabilization and the community at the end of the treatment within each treatment. Pairwise Wilcoxon rank sum test with multiple comparison correction: ^*^P < 0.05, ^*^^*^P < 0.01, ^*^^*^^*^P < 0.005, ^*^^*^^*^^*^P < 0.001.

Weighted Jaccard PCoA indicated that supplementation with scGOS/lcFOS and inulin, irrespective of iron addition, induced a stronger significant shift in overall composition in D1, D2, and D3 microbiota compared to acacia gum, which was driven by the genus *Bifidobacterium* (Fig. 3A). We also observed that changes in microbiota composition upon treatment were very consistent between the two treatment periods within each modelled microbiota (Fig. 3B). Iron supplementation did not induce an overall shift in microbiota composition except in D3 (Fig. 3B) (PERMANOVA, *P* = 0.043 and *P* = 0.0082 in periods 1 and 2, respectively). No significant treatment-induced shift in overall microbiota composition was detected for D4 microbiota. Neither iron nor any of the fiber supplementations resulted in significantly changes in alpha diversity-based ASV richness and Pielou’s evenness when compared to the distance between treatment and stabilization phases of the non-treated IR (Fig. S4).

**Figure 4:**
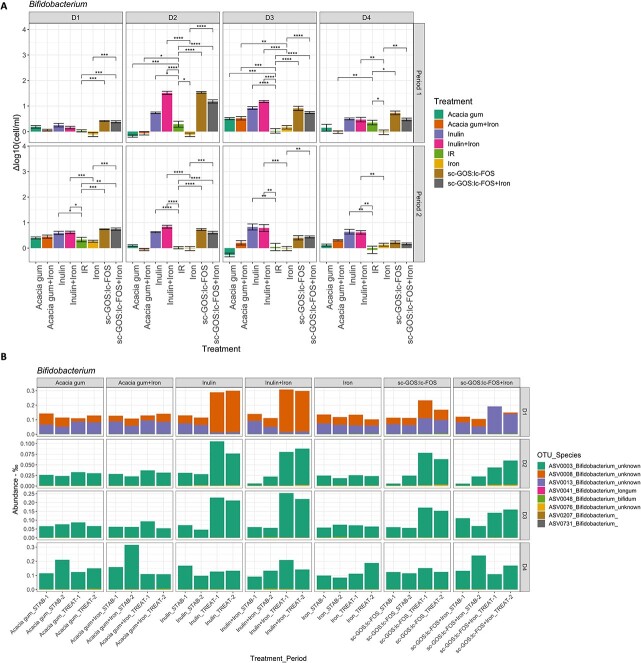
Bifidogenic effect of the treatments. (A) Changes in *Bifidobacterium* concentration (delta log treatment versus stabilization) (cells/ml); qPCR upon treatments across all four donor *in vitro* microbiota. (B) *Bifidobacterium* ASV response upon treatments across all four donor *in vitro* microbiota. ASV taxonomic assignment given in Table S4. Pairwise Wilcoxon rank sum test with multiple comparison correction: ^*^P < 0.05, ^*^^*^P < 0.01, ^*^^*^^*^P < 0.005, ^*^^*^^*^^*^P < 0.001.

The bifidogenic effect from scGOS/lcFOS and/or inulin when supplemented alone or when combined with iron was confirmed by qPCR in all four *in vitro* donor microbiota, resulting in increased *Bifidobacterium* concentrations after fiber supplementation compared to the stabilization period (Fig. 4A). The concentration of *Bifidobacterium* increased the most in D2 microbiota after fiber supplementation (e.g. +0.72 ± 0.05 log for inulin and + 1.28 ± 0.03 log for inulin with iron). Acacia gum only impacted *Bifidobacterium* growth in D3 microbiota. Supplementation with iron alone did not result in an overall decrease in *Bifidobacterium*, though a decrease was observed in one replicate treatment in each of the D2 and D4 microbiota. We observed that *Bifidobacterium* ASV0003 (closest assigned to *Bifidobacterium breve*, *Bifidobacterium scaligerum*, and *Bifidobacterium longum* subsp. *infantis*) was dominant in three donors (D2, D3, D4), while ASV0008 (closest to *Bifidobacterium adolescentis*) and ASV0013 (closest to *Bifidobacterium callitrichidarum, Bifidobacterium catenulatum, Bifidobacterium gallicum, and Bifidobacterium pseudocatenulatum*) dominated in D1 (Fig. 4B and Table S4). Bifidogenic effects of both scGOS/lcFOS and inulin induced an increase of the most abundant *Bifidobacterium* ASVs in D1, D2, and D3 compared to the levels observed before supplementation. In contrast, when scGOS/lcFOS was supplemented during iron treatment in D1, only the relative abundance of ASV0013 increased in both periods, which suggests an iron-induced change in bifidogenic effect in D1 microbiota.

**Figure 5:**
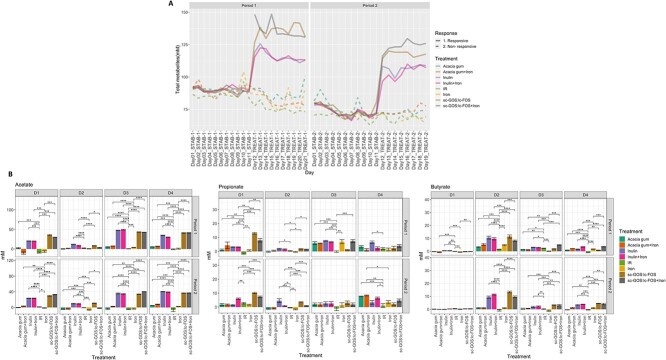
SCFA-promoting effect of the treatments. (A) Total metabolites (mM) over experiment course in representative *in vitro* microbiota D1. (B) Changes in production acetate, propionate, and butyrate (mM) upon treatments across all four donor *in vitro* microbiota (pairwise Wilcoxon rank sum test with multiple comparison correction: ^*^P < 0.05, ^*^^*^P < 0.01, ^*^^*^^*^P < 0.005, ^*^^*^^*^^*^P < 0.001).

The prebiotics scGOS/lcFOS as well as inulin, both with and without iron, showed an evident increase in production of total fermentation metabolites upon initial treatment when compared to non-treated IR and the treatments iron and acacia gum (Fig. 5A and Fig. S5). Iron treatment did not result in a significant or consistent alteration in acetate levels in all four microbiota in both treatment periods. In contrast, supplementation with scGOS/lcFOS and inulin resulted in a strong acetogenic response, with increased acetate up to +40 mM for D1, D3, D4 and up to +15 mM in D2 *in vitro* microbiota (Fig. 5B). Butyrate production was consistently increased upon supplementation with scGOS/lcFOS and inulin, independent of iron supplementation, in D2 (+9 to +13 mM), D3 (+2 to +3 mM) and D4 (+0.8 to +4.5 mM) microbiota (Fig. 5B). Increased propionate production was observed for all treatments across the different donors, with the largest effect for scGOS/lcFOS in D1 microbiota (e.g. +13.0 ± 1.7 mM for scGOS/lcFOS and + 8.3 ± 3.7 mM for scGOS/lcFOS with iron in period 1) (Fig. 5B). The intermediate metabolites formate or lactate increased upon scGOS/lcFOS and inulin supplementation with and without iron in D1, D3 and D4 *in vitro* microbiota (up to +11 mM for formate with scGOS/lcFOS in D3 and up to +15 mM for lactate with inulin in D1) (Fig. S6).

### Donor- and prebiotic-specific changes in opportunistic enteropathogen concentration

**Figure 6:**
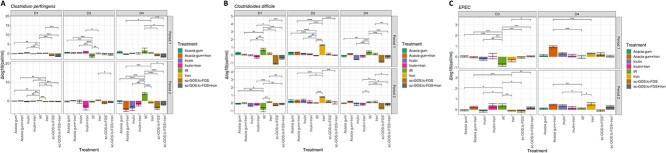
Changes in opportunistic enteropathogens. Bacterial counts (log(cells/ml)) of (A) *Clostridium perfringens*, (B) *Clostridioides difficile*, and (C**)** EPEC upon treatments across all four donor *in vitro* microbiota (pairwise Wilcoxon rank sum test with multiple comparison correction: ^*^P < 0.05, ^*^^*^P < 0.01, ^*^^*^^*^P < 0.005,^*^^*^^*^^*^P < 0.001).

Supplementation with scGOS/lcFOS resulted in a consistent decrease of *C. perfringens* in D1 and D4 microbiota under both iron-supplemented and iron-limited conditions when compared to IR and iron treatment (Fig. 6A). With acacia gum and inulin, the detected decreases were low (D1) or were not replicated in both treatment periods (D4). Iron supplementation resulted in a significant increase in *C. difficile* concentrations in D2 microbiota (+ 1.0 ± 0.3 log), which was not the case when fibers were co-supplemented (Fig. 6B). In D1 and D4 microbiota, supplementation with inulin and particularly scGOS/lcFOS resulted in lower *C. difficile* concentrations. The treatment effect on EPEC concentrations was variable across fiber supplementations; only a consistent increase upon supplementation with acacia gum combined with iron was observed in Donor 4 microbiota (Fig. 6C).

### Donor-microbiota dependent taxonomic response to fiber treatments

**Figure 7:**
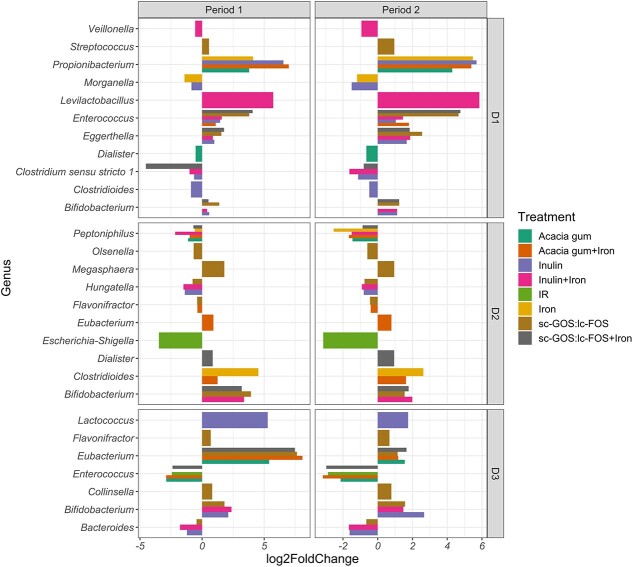
Donor–microbiota dependent taxonomic response. Differentially abundant genera bar plot visualization of DESeq2 outcome. Significant shifts (treatment vs. stabilization) upon treatments in genera relative abundance within TRs compared to IR microbiota in Period 1 and Period 2. The relative abundance and log2FoldChange is given in Table S5.

We next assessed the differential response of gut microbiota by 16S RNA sequencing by comparing the different treatments to non-treated IR microbiota with DESeq2 analysis. Only the genera with both significant and consistent log2 fold changes (FC) between treatment and stabilization in both periods are presented (Fig. 7, Table S5).

Overall, supplementation with iron had a limited effect on the genus relative abundance, with an increase in low relative abundant *Propionibacterium* (log_2_ FC +4.8 ± 1, to 0.10%) and a decrease in *Morganella* (log_2_ FC –1.3 ± 0.2, to 0.47%) in D1 microbiota, and a decrease in low relative abundant *Peptoniphilus* (log_2_ FC –1.6 ± 1.4, to 0.39%) in D2 microbiota. No change in *Enterobacteriaceae* genera was observed in any of the investigated donor microbiota. In line with qPCR results in D2 microbiota, an increased relative abundance of *Clostridioides* (log_2_ FC +3.6 ± 1.3, from 0.02% to 0.33%) was observed after iron supplementation (Fig. 7, Table S5).

Genus relative abundance variations were mainly observed in response to the fiber supplementation and were mostly donor dependent. In D1 microbiota, scGOS/lcFOS as well as inulin supplemented alone or with iron, resulted in an increased relative abundance of *Enterococcus* (4.2 ± 0.6; 4.4 ± 0.5; 1.2 ± 0.3; and 1.5 ± 0.1 log_2_ fold-increase respectively) and *Eggerthella* (2.1 ± 0.7; 1.8 ± 0.1; 1.3 ± 0.5; and 1.4 ± 0.7 log_2_ fold-increase respectively). In D2 microbiota, inulin supplementation with and without iron resulted in lower relative abundance of *Hungatella* (1.1 ± 0.4 and 1.2 ± 0.4 log_2_ fold-decrease respectively). Increased relative abundance of *Eubacterium* was observed in D3 microbiota as a response to supplementation with scGOS/lcFOS with and without iron (4.6 ± 4.1 and 4.4 ± 4.6 log_2_ fold-increases, respectively) and with acacia gum with and without iron (4.6 ± 4.9 and 3.5 ± 2.7 log_2_ fold-increases, respectively). Also, the relative abundance of *Enterococcus* decreased with scGOS/lcFOS along with iron (2.7 ± 0.4 log_2_ fold-decrease) and with acacia gum with and without iron (3 ± 0.2 and 2.9 ± 2.1 log_2_ fold-decrease respectively). Further, inulin supplementation with and without iron in D3 microbiota led to a decrease in *Bacteroides* relative abundance (1.7 ± 0.1 and 1.4 ± 0.3 log_2_ fold-decreases, respectively) (Fig. 7, Table S5).

We also observed genus increases upon fiber supplementation when no iron was co-supplemented such as upon scGOS/lcFOS supplementation in *in vitro* microbiota of D1 (*Streptococcus,* from 22.4% to 26.3%), D2 (*Megasphaera,* from 7.4% to 15.3%) and D3 (*Flavonifractor,* from 5.1% to 6.9% and *Collinsella,* from 16.3% to 24.7%), or upon inulin supplementation in D3 microbiota (*Lactococcus,* from 3.2% to 14.8%). On the other hand, iron supplementation in combination with scGOS/lcFOS enhanced *Dialister* in D2, or iron with inulin enhanced *Levilactobacillus* in D1 microbiota; these increases were not observed when the fibers were added alone. In line with the beta diversity data, no consistent significant taxonomic shifts were detected in D4 microbiota in the two treatment periods.

### Dose–response effects for inulin

**Figure 8:**
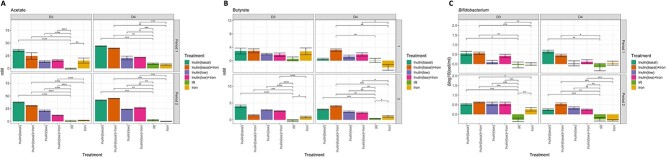
Dose–response assessment. (A) Changes in acetate and butyrate production (mM) upon supplementation with inulin at low and high doses with or without iron across two donors’ *in vitro* microbiota. (B) Changes in bifidobacteria concentration (log(cells/ml)). Pairwise Wilcoxon rank sum test with multiple comparison correction: ^*^P < 0.05, ^*^^*^P < 0.01, ^*^^*^^*^P < 0.005,^*^^*^^*^^*^P < 0.001.

Inulin was selected to determine whether a 50% lower dose still stimulated SCFA production and growth of *Bifidobacterium* in D3 and D4 *in vitro* microbiota. Both the basal and 50% lower inulin dose resulted in a reproducible acetogenic effect in both tested microbiota (Fig. 8A). The mean increase in acetate production upon the lower inulin dose (+ 15 mM for D3 and + 22 mM for D4) was half as high compared to the basal inulin dose (+ 31 mM for D3 and + 45 mM for D4), showing a clear dose–response for inulin on acetate production. Butyrate production was enhanced upon the 50% inulin dose in both microbiota that were tested in Period 2, but to a lesser extent than the 100% dose. The 50% lower inulin dose resulted in a bifidogenic effect, but only in period 2 in both microbiota and thus was less effective in promoting *Bifidobacterium* growth compared to the basal inulin dose (Fig. 8B). The latter was reflected in the overall microbiota response, as a reproducible change in weighted Jaccard distance from the non-treated microbiota was only observed for the basal and not for the lower inulin dose (Fig. S7).

## Discussion

In this *in vitro* study, we compared the prebiotic potential of three different fibers alone and in combination with iron in four different gut microbiota of Kenyan infants living in low hygiene environments and at high risk of developing anemia during weaning.

Our first objective was to induce an alteration of the gut microbiome with iron supplementation. However, on our *in vitro* model, iron supplementation at 12.5 mg iron/day did not impact the *Enterobacteriaceae* abundance or the overall gut microbiota profile*,* which contradicts previous *in vivo* findings [11–13]. Similar results were seen in children populations with low enteropathogen levels (50 mg iron/day, [41]) and in *in vitro* studies with child microbiota (30.4 mg iron/day, [42]). In healthy adult human fecal microbiota, 3.4 mg/day of iron led to minimal, donor-specific microbiota changes without increased *Enterobacteriaceae* levels [43]. Our study differs from previous *in vitro* studies, as we investigated African infant gut microbiota containing several naturally-occurring opportunistic enteropathogens. Our data suggest that the *in vivo* observed iron-induced microbiota dysbiosis may be due to an iron-host–microbiota interaction instead of a direct iron-microbiota interaction.

We observed that iron supplementation *in vitro* resulted in increased concentration and relative abundance of *Clostridioides difficile* in one microbiota. Such an increase was also observed in previous trials with Kenyan infants aged 6–10 months after iron supplementation with [44] or without [11] antibiotics. In line with this, a recent study demonstrated that iron (Fe3+, which is also the form expected in our *in vitro* model at pH 5.8) stimulated the growth of *C. difficile* in a dose-dependent manner and consequently led to higher levels of Toxin A, necessary for pathogenesis, after iron supplementation [45].

In our model, scGOS/lcFOS and inulin inhibited growth of *C. difficile* and *C. perfringens* in two of the four *in vitro* microbiota both during and without iron supplementation, which is consistent with studies in European infants supplemented with the same prebiotics [46–48]. Decreased levels of enteropathogens, concomitant with a pronounced increase in SCFA production, were measured upon supplementation with scGOS/lcFOS and inulin with iron. We anticipate that this effect could be stronger *in vivo* because the resulting lower pH may further inhibit the growth of enteropathogens. Additionally, SCFA also inhibit the expression of their virulence genes [49]. A lower fecal pH was indeed detected in European infant studies involving formula supplemented with scGOS/lcFOS or inulin [27, 48, 50].

Supplementation with scGOS/lcFOS and native inulin strongly enhanced production of acetate (up to 2-fold) and the growth of *Bifidobacterium* during iron supplementation in all studied donor microbiota, despite their baseline compositional and functional differences. This is consistent with previous studies in European infants who received the same scGOS/lcFOS (9:1 ratio) [27, 47, 51] or with *in vitro* batch incubations with European fecal infant microbiota supplemented with native inulin [29, 52]. We observed that a 50% lower dose of native inulin equally promoted acetate production and *Bifidobacterium* growth, but its overall effect on the microbial community composition was lower compared to the 100% dose, which suggests that higher doses may be needed to induce community changes. The fiber or prebiotic dose–response effect on the gut microbiome is not well studied in infants. In adults, trials with distinct dietary fibers showed that the dominant effects were dose-dependent and plateaued at 35 g/day [53].

While *Bifidobacterium* exhibited convergent response across donors, other taxa had variable responses to fiber treatments during and without iron supplementation. This emphasizes the importance of considering individual responses when assessing the impact of prebiotics on gut microbiota as previously observed for adult microbiota [53–55]. In our study, scGOS/lcFOS demonstrated a butyrogenic effect alongside a reduced acetogenic response in the D2 microbiota, suggesting potential cross-feeding interactions between different bacterial groups, possibly involving acetate- to butyrate-producing taxa or lactate- to butyrate-producing taxa [56]. *Megasphaera*, containing butyrate-producing species, was notably stimulated by scGOS/lcFOS supplementation in D2 microbiota. Previously, cross-feeding between FOS-induced lactate and butyrate production by *Megasphaera elsdenii* was demonstrated in an animal model [57]. Also, other taxa from the detected genera *Eubacterium*, *Hungatella*, *Flavonifractor*, and *Lachnoclostridium* could have contributed to the increased butyrate production in D2 microbiota. Next, *Eggerthella,* an acetate-, lactate-, and succinate-producing gut commensal, increased with scGOS/lcFOS and inulin in D1 microbiota and could explain the observed increase in lactate in this system. Increased relative abundance of *Eubacterium* in microbiota supplemented with scGOS/lcFOS and acacia gum was only observed in D3 microbiota, and was previously detected in rats upon GOS supplementation [58]. Inulin supplementation led to lower *Bacteroides* relative abundance in D2 microbiota, potentially due to the competitive advantage of inulin-degrading *Bifidobacterium* taxa. Our *in vitro* finding supports the negative association detected between *Bacteroides fragilis* and two *Bifidobacterium* species in fecal metagenomes from the same Kenyan infant population [59].

The emerging prebiotic candidate acacia gum induced little to no effect on the model African infant gut microbiota in contrast to the established prebiotics scGOS/lcFOS and inulin. The chemical structure of acacia gum is more complex and its degradation requires different bacterial glycoside hydrolases (GH) from different carbohydrate-active enzyme families including GH16: endo-β-1,3-galactanase, GH43: exo- β-1,3-galactanase, GH154/GH105: β-glucuronidase, GH145/PL27: α-L-rhamnosidase/L-rhamnose-α-1,4-D-glucuronate lyase, and GH27/GH97: β-L-arabinopyranosidase/ α-galactosidase [60]. Moreover, *B. longum* subsp. *longum* was recently identified as a specialist degrader of acacia gum, because of its enzyme 3-O-α-D-galactosyl-α-L-arabinofuranosidase (GH39) [61]. Acacia gum-degrading taxa could be absent or at low prevalence in the gut microbiota of partially breast-fed Kenyan infants aged 6–10 months. This was indeed confirmed in a recently published metagenomic study of fecal samples of the same Kenyan infant population, which detected genes for only three of the five bacterial enzymes for acacia gum degradation (GH43, GH16, and GH27) at low gene prevalence and abundance [59]. Previously published bifidogenic and SCFA-promoting effects of acacia gum were mainly reported in studies with human adult feces [32, 34, 60]; *in vitro* studies involving American infant feces also did not detect acacia gum fermentation [62, 63]. Therefore, our study highlights the importance of evaluating established prebiotics and emerging prebiotic candidates in the target host microbiota. In this case, there is a marked difference in functional capacity and phylogenetic composition between Western and rural African infants, which are still insufficiently characterized [64].

We conclude that both scGOS/lcFOS and native inulin, but not acacia gum, have strong prebiotic potential in Kenyan infant gut microbiota during iron supplementation by promoting growth of beneficial gut bacteria including bifidobacteria and inhibiting enteropathogen growth by enhanced SCFA production. Both fibers promoted butyrate or propionate production in a microbiota-specific way. Acacia gum had little impact on fermentation and microbiota composition and therefore has low prebiotic potential in Kenyan infants at weaning age. This study demonstrates the importance of comparing prebiotics and other fibers in the target population *in vitro* prior to *in vivo* clinical studies. Future clinical trials with iron supplementation in combination with scGOS/lcFOS or native inulin in African infants are needed to assess their prebiotic effect and impact on iron absorption.

## Supplementary Material

Momo-Cabrera_SupplementaryInfo_ycae033

## Data Availability

The sequencing datasets generated during and/or analyzed during the current study are available in the European Nucleotide Archive (ENA) repository (https://www.ebi.ac.uk/ena/browser/view/PRJEB67393). The other data are available from the corresponding author on reasonable request.

## References

[1] Kassebaum NJ , Jasrasaria R, Naghavi M et al. A systematic analysis of global anemia burden from 1990 to 2010. Bloo*d* 2014;123:615–24. 10.1182/blood-2013-06-508325 24297872 PMC3907750

[2] WHO . The Global Prevalence of Anaemi*a*. Geneva: World Health Organization, 2015.

[3] Gedfie S , Getawa S, Melku M. Prevalence and associated factors of iron deficiency and iron deficiency anemia among u1nder-5 children: a systematic review and meta-analysis. Glob Pediatr Hea*l* 2022;9:2333794X2211108. 10.1177/2333794X221110860 PMC9272181 35832654

[4] Paganini D , Uyoga MA, Cercamondi CI et al. Consumption of galacto-oligosaccharides increases iron absorption from a micronutrient powder containing ferrous fumarate and sodium iron EDTA: a stable-isotope study in Kenyan infants. Am J Clin Nut*r* 2017;106:1020–31. 10.3945/ajcn.116.145060 28814396

[5] Giorgetti A , Paganini D, Nyilima S et al. The effects of 2′-fucosyllactose and lacto-N-neotetraose, galacto-oligosaccharides, and maternal human milk oligosaccharide profile on iron absorption in Kenyan infants. Am J Clin Nut*r* 2023;117:64–72. 10.1016/j.ajcnut.2022.10.005 36789945

[6] De-Regil LM , Suchdev PS, Vist GE et al. Home fortification of foods with multiple micronutrient powders for health and nutrition in children under two years of age (review). Evid Based Child Healt*h* 2013;8:112–201. 10.1002/ebch.1895 23878126

[7] WHO . Use of Multiple Micronutrient Powders for Point-of-Use Fortification of Foods Consumed by Infants and Young Children Aged 6–23 Months and Children Aged 2–12 year*s*. Geneva: World Health Organization; 2016. Licence: CC BY-NC-SA 3.0 IGO. 28079999

[8] Paganini D , Zimmermann MB. The effects of iron fortification and supplementation on the gut microbiome and diarrhea in infants and children: a review. Am J Clin Nut*r* 2017;106:1688S–93. 10.3945/ajcn.117.156067 29070552 PMC5701709

[9] Tondeur MC , Schauer CS, Christofides AL et al. Determination of iron absorption from intrinsically labeled microencapsulated ferrous fumarate (sprinkles) in infants with different iron and hematologic status by using a dual-stable-isotope method. Am J Clin Nut*r* 2004;80:1436–44. 10.1093/ajcn/80.5.1436 15531698

[10] Puga AM , de L Samaniego-Vaesken M, Montero-Bravo A et al. Iron supplementation at the crossroads of nutrition and gut microbiota: the state of the art. Nutrient*s* 2022;14:1926. 10.3390/nu14091926 PMC9102039 35565894

[11] Jaeggi T , Kortman GAM, Moretti D et al. Iron fortification adversely affects the gut microbiome, increases pathogen abundance and induces intestinal inflammation in Kenyan infants. Gu*t* 2015;64:731–42. 10.1136/gutjnl-2014-307720 25143342

[12] Paganini D , Uyoga MA, Kortman GAM et al. Prebiotic galacto-oligosaccharides mitigate the adverse effects of iron fortification on the gut microbiome: a randomised controlled study in Kenyan infants. Gu*t* 2017;66:1956–67. 10.1136/gutjnl-2017-314418 28774885

[13] Tang M , Frank DN, Hendricks AE et al. Iron in micronutrient powder promotes an unfavorable gut microbiota in Kenyan infants. Nutrient*s* 2017;9:1–12. 10.3390/nu9070776 PMC5537890 28753958

[14] Popovic A , Bourdon C, Wang PW et al. Micronutrient supplements can promote disruptive protozoan and fungal communities in the developing infant gut. Nat Commu*n* 2021;12:1–13. 10.1038/s41467-021-27010-3 34795270 PMC8602372

[15] Gozzelino R , Arosio P. Iron homeostasis in health and disease. Int J Mol Sc*i* 2016;17:2–14. 10.3390/ijms17010130 PMC4730371 26805813

[16] Lopez CA , Skaar EP. The impact of dietary transition metals on host-bacterial interactions. Cell Host Microb*e* 2018;23:737–48. 10.1016/j.chom.2018.05.008 29902439 PMC6007885

[17] Dostal A , Chassard C, Hilty FM et al. Iron depletion and repletion with ferrous sulfate or electrolytic iron modifies the composition and metabolic activity of the gut microbiota in rats. J Nut*r* 2012;142:271–7. 10.3945/jn.111.148643 22190022 PMC3260059

[18] Dostal A , Lacroix C, Pham VT et al. Iron supplementation promotes gut microbiota metabolic activity but not colitis markers in human gut microbiota-associated rats. Br J Nut*r* 2014;111:2135–45. 10.1017/S000711451400021X 24555487

[19] Dostal A , Lacroix C, Bircher L et al. Iron modulates butyrate production by a child gut microbiota in vitro. MBi*o* 2015;6:e01453–15. 10.1128/mBio.01453-15 26578675 PMC4659462

[20] Naikare H , Palyada K, Panciera R et al. Major role for FeoB in campylobacter jejuni ferrous iron acquisition, gut colonization, and intracellular survival. Infect Immu*n* 2006;74:5433–44. 10.1128/IAI.00052-06 16988218 PMC1594910

[21] Yu X , Gan Z, Wang Z et al. Increased iron availability aggravates the infection of *Escherichia coli* O157:H7 in mice. Biol Trace Elem Re*s* 2019;190:457–65. 10.1007/s12011-018-1579-4 30456562

[22] Huynh U , Zastrow ML. Metallobiology of *Lactobacillaceae* in the gut microbiome. J Inorg Bioche*m* 2023;238:112023. 10.1016/j.jinorgbio.2022.112023 36270041 PMC9888405

[23] Vazquez-Gutierrez P , de Wouters T, Werder J et al. High iron-sequestrating bifidobacteria inhibit enteropathogen growth and adhesion to intestinal epithelial cells in vitro. Front Microbio*l* 2016;7:7. 10.3389/fmicb.2016.01480 27713730 PMC5031772

[24] Gibson GR , Hutkins R, Sanders ME et al. Expert consensus document: the international scientific Association for Probiotics and Prebiotics (ISAPP) consensus statement on the definition and scope of prebiotics. Nat Rev Gastroenterol Hepato*l* 2017;14:491–502. 10.1038/nrgastro.2017.75 28611480

[25] Slavin J . Fiber and prebiotics: mechanisms and health benefits. Nutrient*s* 2013;5:1417–35. 10.3390/nu5041417 23609775 PMC3705355

[26] Davani-Davari D , Negahdaripour M, Karimzadeh I et al. Prebiotics: Definition, Types, Sources, Mechanisms, and Clinical application*s*, Vol. 8. MDPI: Foods, 2019. 10.3390/foods8030092 PMC6463098 30857316

[27] Knol J , Scholtens P, Kafka C et al. Colon microflora in infants fed formula with galacto- and fructo-oligosaccharides: more like breast-fed infants. J Pediatr Gastroenterol Nut*r* 2005;40:36–42. 15625424 10.1097/00005176-200501000-00007

[28] Borewicz K , Suarez-Diez M, Hechler C et al. The effect of prebiotic fortified infant formulas on microbiota composition and dynamics in early life. Sci Re*p* 2019;9:1–13. 10.1038/s41598-018-38268-x 30792412 PMC6385197

[29] Logtenberg MJ , Akkerman R, An R et al. Fermentation of chicory fructo-oligosaccharides and native inulin by infant fecal microbiota attenuates pro-inflammatory responses in immature dendritic cells in an infant-age-dependent and fructan-specific way. Mol Nutr Food Re*s* 2020;64:e2000068. 10.1002/mnfr.202000068 32420676 PMC7378940

[30] De Filippo C , Di Paola M, Ramazzotti M et al. Diet, environments, and gut microbiota. A preliminary investigation in children living in rural and urban Burkina Faso and Italy. Front Microbio*l* 2017;8:1979. 10.3389/fmicb.2017.01979 PMC5645538 29081768

[31] Wyatt GM , Bayliss CE, Holcroft JD. A change in human faecal flora in response to inclusion of gum arabic in the diet. Br J Nut*r* 1986;55:261–6. 10.1079/BJN19860033 2823865

[32] Michel C , Kravtchenko TP, David A et al. In vitro prebiotic effects of acacia gums onto the human intestinal microbiota depends on both botanical origin and environmental pH. Anaerob*e* 1998;4:257–66. 10.1006/anae.1998.0178 16887651

[33] Bliss DZ , Weimer PJ, Jung HJG et al. In vitro degradation and fermentation of three dietary fiber sources by human colonic bacteria. J Agric Food Che*m* 2013;61:4614–21. 10.1021/jf3054017 23556460 PMC3668776

[34] Calame W , Weseler AR, Viebke C et al. Gum arabic establishes prebiotic functionality in healthy human volunteers in a dose-dependent manner. Br J Nut*r* 2008;100:1269–75. 10.1017/S0007114508981447 18466655

[35] Cherbut C , Michel C, Raison V et al. Acacia gum is a bifidogenic dietary fibre with high digestive tolerance in healthy humans. Microb Ecol Health Di*s* 2003;15:43–50. 10.1080/08910600310014377

[36] Isenring J , Bircher L, Geirnaert A et al. In vitro human gut microbiota fermentation models: opportunities, challenges, and pitfalls. Microbiome Res Report*s* 2023;2:2. 10.20517/mrr.2022.15 PMC10688811 38045607

[37] Rachmühl C , Lacroix C, Giorgetti A et al. Validation of a batch cultivation protocol for fecal microbiota of Kenyan infants. BMC Microbio*l* 2023;23:174. 10.1186/s12866-023-02915-9 37403024 PMC10318780

[38] Rachmühl C , Lacroix C, Cabrera PM et al. Long-term continuous cultivation of Kenyan infant fecal microbiota using the host adapted PolyFermS model. Sci Rep. 2023;13:20563. 10.1038/s41598-023-47131-7 PMC10667343 37996456

[39] Heinemann M , Strauchs C, Lütgehetmann M et al. Polymicrobial enteric infections in African infants with diarrhoea—results from a longitudinal prospective case–control study. Clin Microbiol Infec*t* 2021;27:1792–8. 10.1016/j.cmi.2021.03.020 33813114

[40] Hernandez RJ , Gutowski D, Guire KE. Capacity of the colon in children. AJR Am J Roentgeno*l* 1979;133:683–4. 10.2214/ajr.133.4.683 114012

[41] Dostal A , Baumgartner J, Riesen N et al. Effects of iron supplementation on dominant bacterial groups in the gut, faecal SCFA and gut inflammation: a randomised, placebo-controlled intervention trial in south African children. Br J Nut*r* 2014;112:547–56. 10.1017/S0007114514001160 24916165

[42] Dostal A , Fehlbaum S, Chassard C et al. Low iron availability in continuous in vitro colonic fermentations induces strong dysbiosis of the child gut microbial consortium and a decrease in main metabolites. FEMS Microbiol Eco*l* 2013;83:161–75. 10.1111/j.1574-6941.2012.01461.x 22845175 PMC3511601

[43] Celis AI , Relman DA, Huang KC. The impact of iron and heme availability on the healthy human gut microbiome in vivo and in vitro. Cell Chem Bio*l* 2023;30:110–126.e3. 10.1016/j.chembiol.2022.12.001 36603582 PMC9913275

[44] Paganini D , Uyoga MA, Kortman GAM et al. Iron-containing micronutrient powders modify the effect of oral antibiotics on the infant gut microbiome and increase post-antibiotic diarrhoea risk: a controlled study in Kenya. Gu*t* 2019;68:645–53. 10.1136/gutjnl-2018-317399 30448776

[45] Yamaki J , Chawla S, Tong S et al. Iron effects on *Clostridioides difficile* toxin production and antimicrobial susceptibilities. Antibiotic*s* 2022;11:1–13. 10.3390/antibiotics11050537 PMC9137654 35625180

[46] Knol J , Boehm G, Lidestri M et al. Increase of faecal bifidobacteria due to dietary oligosaccharides induces a reduction of clinically relevant pathogen germs in the faeces of formula-fed preterm infants. Acta Paediatr Int J Paediatr Supp*l* 2005;94:31–3. 10.1111/j.1651-2227.2005.tb02152.x 16214763

[47] Scholtens PAMJ , Alliet P, Raes M et al. Fecal secretory immunoglobulin A is increased in healthy infants who receive a formula with short-chain galacto-oligosaccharides and long-chain fructo-oligosaccharides. J Nut*r* 2008;138:1141–7. 10.1093/jn/138.6.1141 18492847

[48] Huet F , Abrahamse-Berkeveld M, Tims S et al. Partly fermented infant formulae with specific oligosaccharides support adequate infant growth and are well-tolerated. J Pediatr Gastroenterol Nut*r* 2016;63:e43–53. 10.1097/MPG.0000000000001360 27472478 PMC5051523

[49] Lackraj T , Kim JI, Tran SL et al. Differential modulation of flagella expression in enterohaemorrhagic *Escherichia coli* O157: H7 by intestinal short-chain fatty acid mixes. Microbiol (United Kingdom*)* 2016;162:1761–72. 10.1099/mic.0.000357 27535670

[50] Oswari H , Widodo AD, Handayani F et al. Dosage-related prebiotic effects of inulin in formula-fed infants. Pediatr Gastroenterol Hepatol Nut*r* 2019;22:63–71. 10.5223/pghn.2019.22.1.63 30671375 PMC6333587

[51] Moro G , Arslanoglu S, Stahl B et al. A mixture of prebiotic oligosaccharides reduces the incidence of atopic dermatitis during the first six months of age. Arch Dis Chil*d* 2006;91:814–9. 10.1136/adc.2006.098251 16873437 PMC2066015

[52] Akkerman R , Logtenberg MJ, Beukema M et al. Chicory inulin enhances fermentation of 2′-fucosyllactose by infant fecal microbiota and differentially influences immature dendritic cell and T-cell cytokine responses under normal and Th2-polarizing conditions. Food Func*t* 2021;12:9018–29. 10.1039/D1FO00893E 34382992

[53] Deehan EC , Yang C, Perez-Muñoz ME et al. Precision microbiome modulation with discrete dietary fiber structures directs short-chain fatty acid production. Cell Host Microb*e* 2020;27:389–404.e6. 10.1016/j.chom.2020.01.006 32004499

[54] Vandeputte D , Falony G, Vieira-Silva S et al. Prebiotic inulin-type fructans induce specific changes in the human gut microbiota. Gu*t* 2017;66:1968–74. 10.1136/gutjnl-2016-313271 28213610 PMC5739857

[55] Baxter NT , Schmidt AW, Venkataraman A et al. Dynamics of human gut microbiota and short-chain fatty acids in response to dietary interventions with three fermentable fibers. MBi*o* 2019;10:e02566-18. 10.1128/mBio.02566-18 PMC6355990 30696735

[56] Rivière A , Selak M, Lantin D et al. Bifidobacteria and butyrate-producing colon bacteria: importance and strategies for their stimulation in the human gut. Front Microbio*l* 2016;7:979. 10.3389/fmicb.2016.00979 27446020 PMC4923077

[57] Hashizume K , Tsukahara T, Yamada K et al. *Megasphaera elsdenii* JCM1772T normalizes hyperlactate production in the large intestine of fructooligosaccharide-fed rats by stimulating butyrate production. J Nut*r* 2003;133:3187–90. 10.1093/jn/133.10.3187 14519808

[58] Hernández-Hernández O , Marín-Manzano MC, Rubio LA et al. Monomer and linkage type of galacto-oligosaccharides affect their resistance to ileal digestion and prebiotic properties in rats. J Nut*r* 2012;142:1232–9. 10.3945/jn.111.155762 22649257

[59] Derrien M , Mikulic N, Uyoga MA et al. Gut microbiome function and composition in infants from rural Kenya and association with human milk oligosaccharides. Gut Microbe*s* 2023;15:2178793. 10.1080/19490976.2023.2178793 PMC9980514 36794816

[60] Fujita K , Sasaki Y, Kitahara K. Degradation of plant arabinogalactan proteins by intestinal bacteria: characteristics and functions of the enzymes involved. Appl Microbiol Biotechno*l* 2019;103:7451–7. 10.1007/s00253-019-10049-0 31384991

[61] Sasaki Y , Horigome A, Odamaki T et al. Novel 3-O-α-d-Galactosyl-α-l-arabinofuranosidase for the assimilation of gum Arabic arabinogalactan protein in *Bifidobacterium longum* subsp. longum. *Appl Environ Microbiol* 2021;87:e02690-20. 10.1128/AEM.02690-20 PMC8117759 33674431

[62] Vester Boler BM , Rossoni Serao MC, Faber TA et al. In vitro fermentation characteristics of select nondigestible oligosaccharides by infant fecal inocula. J Agric Food Che*m* 2013;61:2109–19. 10.1021/jf305056f 23379900

[63] Chow J , Panasevich MR, Alexander D et al. Fecal metabolomics of healthy breast-fed versus formula-fed infants before and during in vitro batch culture fermentation. J Proteome Re*s* 2014;13:2534–42. 10.1021/pr500011w 24628373

[64] Tamburini FB , Maghini D, Oduaran OH et al. Short- and long-read metagenomics of urban and rural south African gut microbiomes reveal a transitional composition and undescribed taxa. Nat Commu*n* 2022;13:926. 10.1038/s41467-021-27917-x 35194028 PMC8863827

