# Supplementary material for: Comparative prebiotic potential of galacto- and fructo-oligosaccharides, native inulin, and acacia gum in Kenyan infant gut microbiota during iron supplementation
Source: ISME Commun. 2024 Mar 11;4(1):ycae033. doi: 10.1093/ismeco/ycae033 (PMC11107946; doi:10.1093/ismeco/ycae033)
Supplement: Momo-Cabrera_SupplementaryInfo_ycae033 [file momo-cabrera_supplementaryinfo_ycae033.docx]

**Supplementary information**

**Title:**

Comparative prebiotic potential of galacto-fructo-oligosaccharides, inulin and acacia gum in Kenyan infant gut microbiota during iron supplementation

**Running title**
Distinct fiber effect in ex vivo Kenyan infant gut microbiota

Paula Momo Cabrera^1^, Carole Rachmühl^1^, Muriel Derrien^2,3^, Raphaëlle Bourdet-Sicard^2^, Christophe Lacroix^1*^, Annelies Geirnaert^1*^

^1^ Laboratory of Food Biotechnology, Institute of Food, Nutrition and Health, ETH Zurich, Zurich, Switzerland

^2^ Danone Global Research & Innovation Center, Gif sur Yvette, France

^3^ Current address: Laboratory of Molecular Bacteriology, Department of Microbiology and Immunology, Rega Institute, KU Leuven, Belgium
^*^ correspondence: annelies.geirnaert@hest.ethz.ch & christophe.lacroix@hest.ethz.ch

**Supplementary methods:**

**Quantitative PCR**

The qPCR analysis was conducted with the SYBR Green PCR Master Mix kit (Bioline GmbH, Germany) in Roche LightCycler^®^ 480 (Hoffmann-La Roche, Basel, Switzerland). Each DNA sample was analyzed in technical triplicates. A standard curve was generated by 10-fold serial dilutions using the linearized plasmid containing each target amplicon. With the LightCycler 480 software the standard curves and the absolute copy number of the samples was calculated with the Absolute Quantification/ 2^nd^ derivative max function, and PCR efficiency (%) was derived from the slope of the standard curve of each qPCR assay. Assays with an efficiency of 80-110% (slope of 3.2-3.9) were included in data analysis. The corresponding bacterial cell number was estimated using the 16S rRNA gene copy number for each specific target according to the Ribosomal RNA database (1).

**Amplicon sequencing and data analysis**

The 515F (5’- GTGCCAGCMGCCGCGGTAA-3’) and 806R (5’-GGACTACHVGGGTWTCTAAT-3’) primers were used to amplify and sequence the V4 hypervariable region of the 16S rRNA and Nextera Index primers were used for barcoding of the amplicons. The amplified fragment with adapters was purified using AMPure XP beads (Beckman Coulter Genomics, CA, USA). Sequencing was performed with Illumina MiSeq with V2 reagent kit for 2 x 250 cycles to generate 250 bp paired-end reads in each direction, and supplemented with 20 % of PhiX. Atropos software was used as a trimming tool to remove Illumina adaptors and gene-specific PCR primers. The DADA2-pipeline was used to generate amplicon sequencing variants (ASVs). Chimeric sequences were identified and removed and taxonomy was assigned using the SILVA database (v.132) as previously described (2).

**Data analysis details**

To analyze qPCR and metabolite data, the difference (delta) between the treatment period (last three days) and previous stabilization period (last three days) was calculated for each treatment within each period (3). In the case of the metabolite data, the difference was calculated from absolute concentrations. In the case of qPCR data, the log difference was calculated using log-transformed concentrations of bacterial targets (log(x_treatment_)-log(x_stabilization_)). The resulting delta values of each treatment (iron, acacia gum± iron, scGOS/lcFOS± iron, and inulin± iron) was compared with that of the non-treated inoculum reactor (IR) to indicate potential treatment effects. Moreover, delta values from iron+fiber were compared to iron to identify additional fiber effects.

Data analysis and visualization of 16S amplicon sequencing was performed using the R packages phyloseq (version 1.40.0), vegan (version 2.6.2), DESeq2 (version 1.36.0) and ggplot2 (version 3.3.6). The statistical analysis was based on the comparison between treatment and respective stabilization phases from each individual period of the 7 treatments and control (IR). Alpha diversity and community similarity (beta diversity) were calculated on rarified data (using the rarefy_even_depth function from the phyloseq package), in which samples with a sequencing depth lower than 1000 reads were omitted. Differential abundance analysis was performed on non-rarefied relative abundance datasets using the Wald test with DESeq2 (4). Only those ASVs present in at least 30% of the tested samples were considered.

**Supplementary Figures**


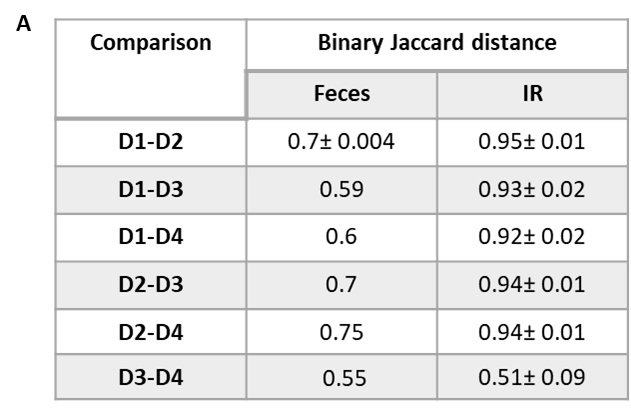

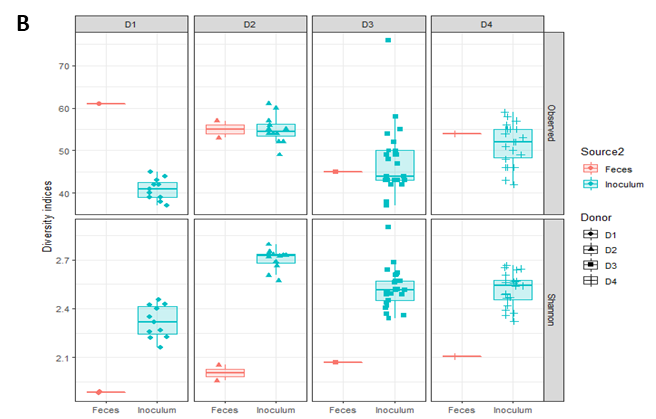


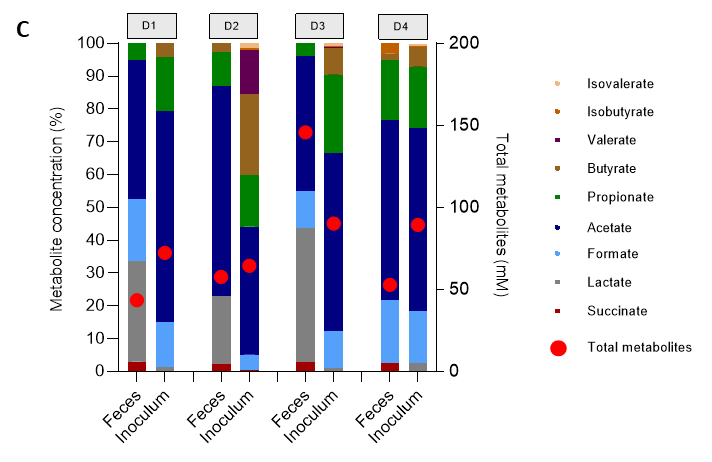


**Figure S1:** **Differences in four donor in vitro baseline microbiota**. **A** Inter-donor Binary Jaccard distances in fecal samples and in modelled microbiota. **B** Alpha diversity in the fecal sample and the corresponding IR at different days of fermentation for all 4 donors (D1, D2, D3 and D4). **C** Metabolite composition of fecal samples and corresponding IR from D1, D2, D3 and D4. The relative proportion of intermediate metabolites, SCFAs and BCFAs are shown on the left y-axis and the total metabolite concentrations are shown on the right y-axis.


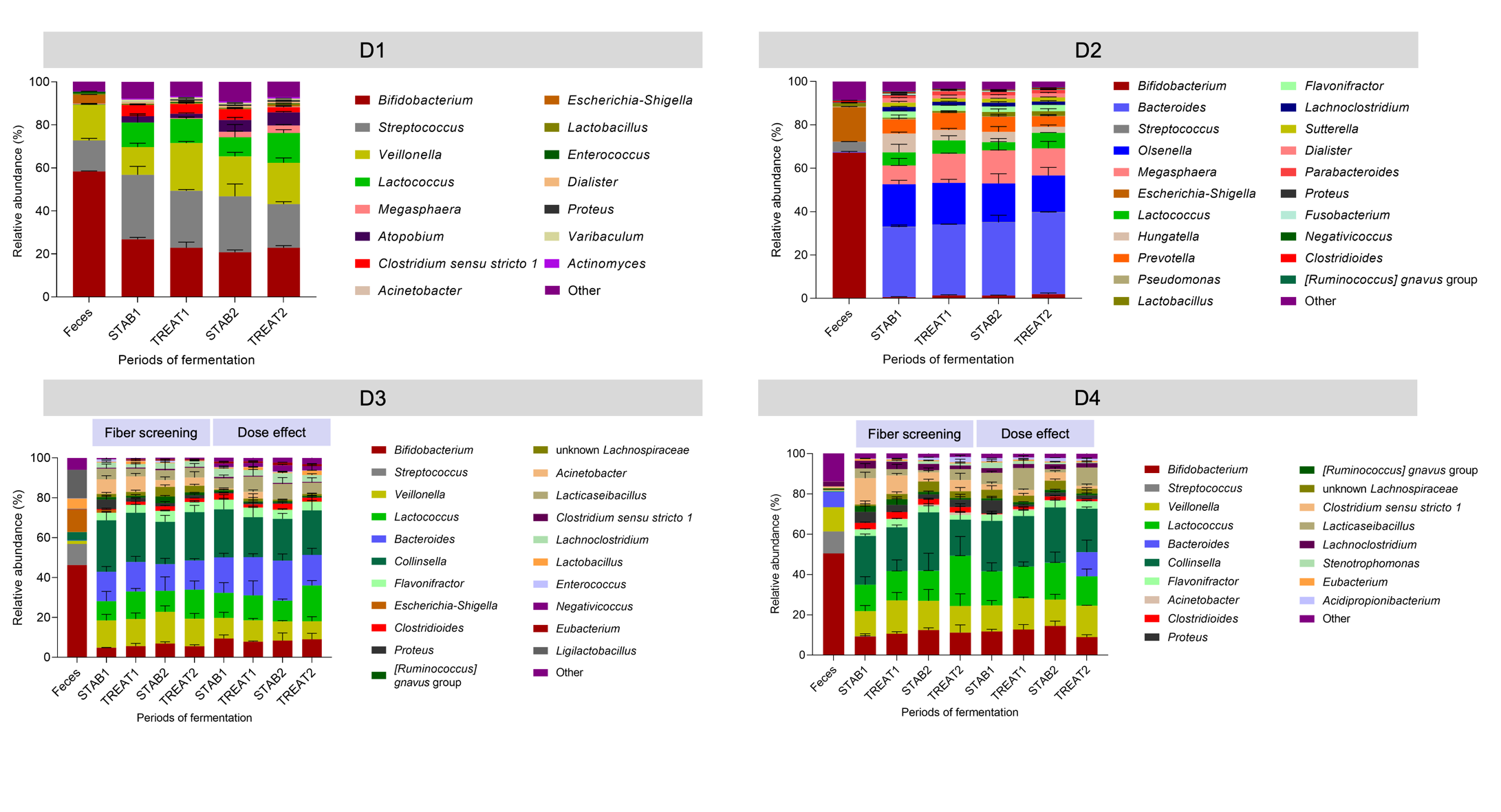
**Figure S2:** **Stability of baseline in vitro microbiota composition.** The relative abundance of top bacterial genera in the fecal samples and the corresponding in vitro PolyFermS IR inoculated with D1, D2, D3 and D4 immobilized fecal microbiota during the last three days of stabilization (STAB) and treatment (TREAT) periods. Means ± standard deviations (SD) of three consecutive fermentation days are shown. Genera representing more than 10% across all samples analyzed are shown in the bar plot.

**
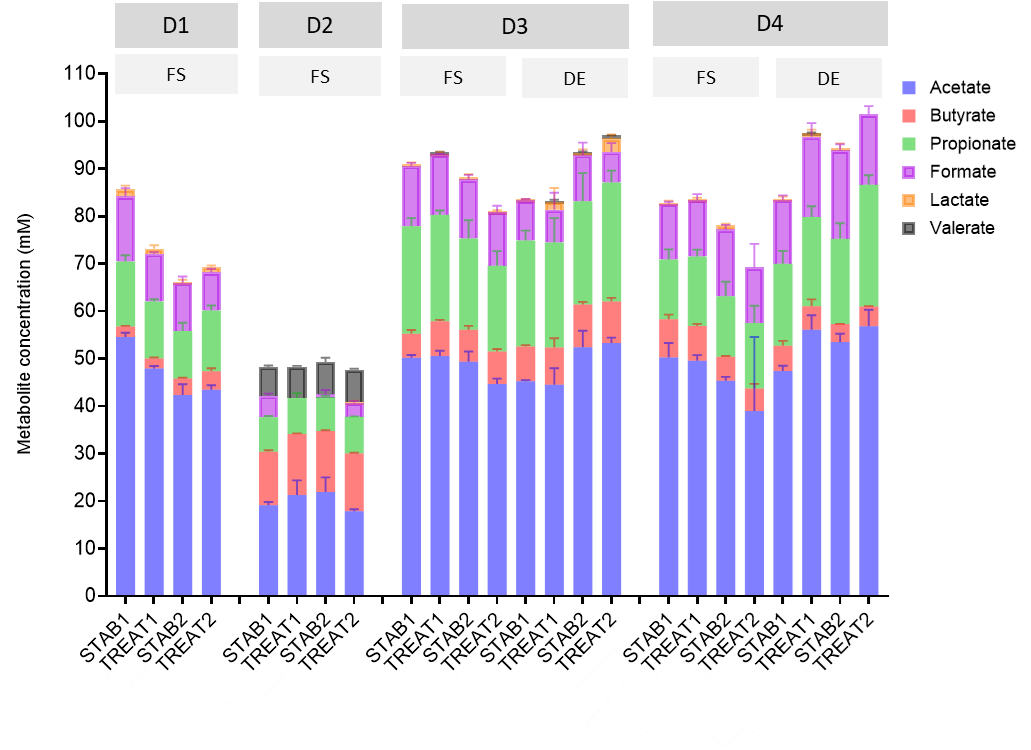
**

**Figure S3:** **Metabolic stability of baseline in vitro microbiota.** The metabolite profile of PolyFermS IR inoculated with D1, D2, D3 and D4 immobilized fecal microbiota during the last three days of stabilization (STAB) and treatment (TREAT) in fiber screening (FS) and dose effect (DE) experimental phases. Means ± standard deviations (SD) of three consecutive fermentation days are shown.

**
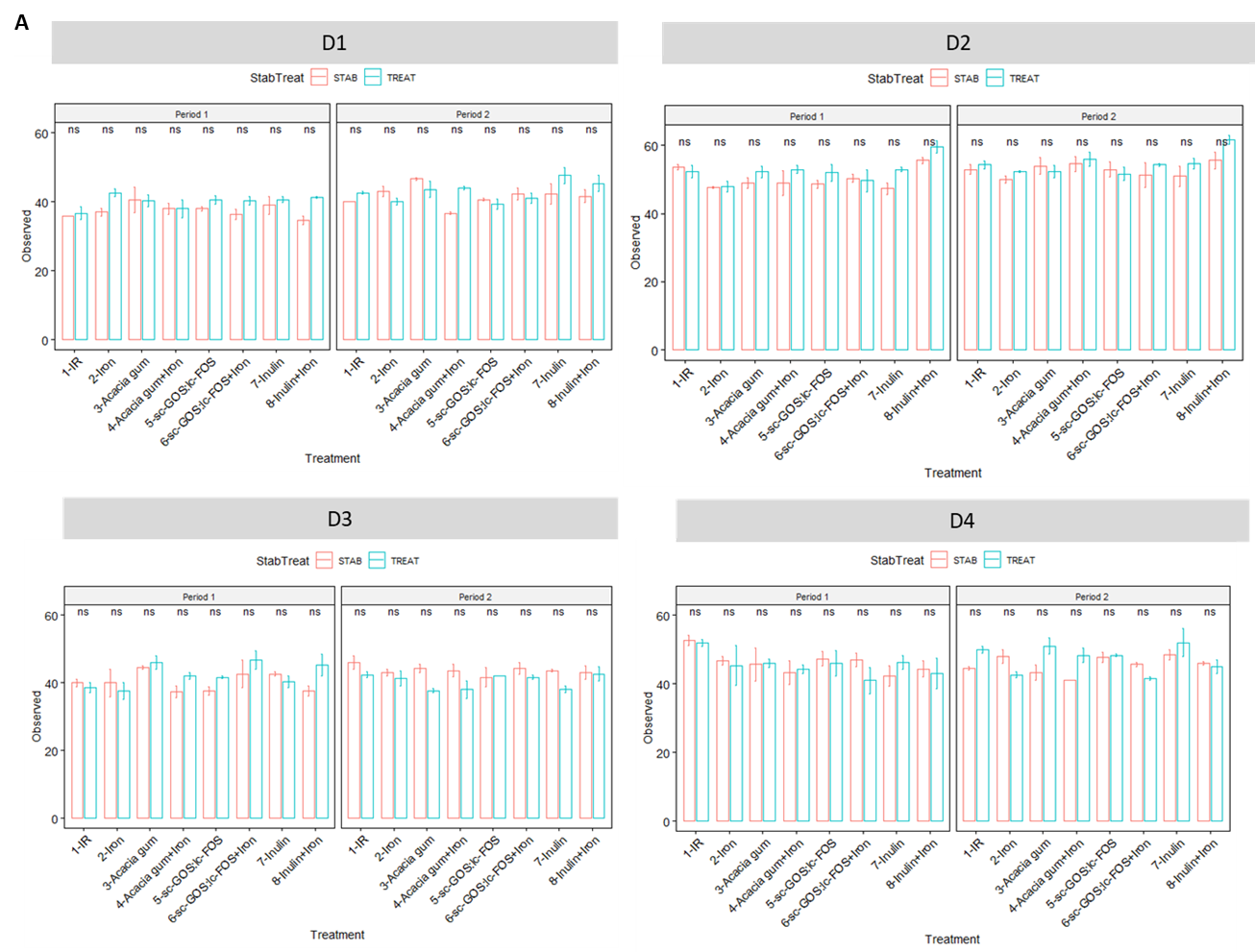

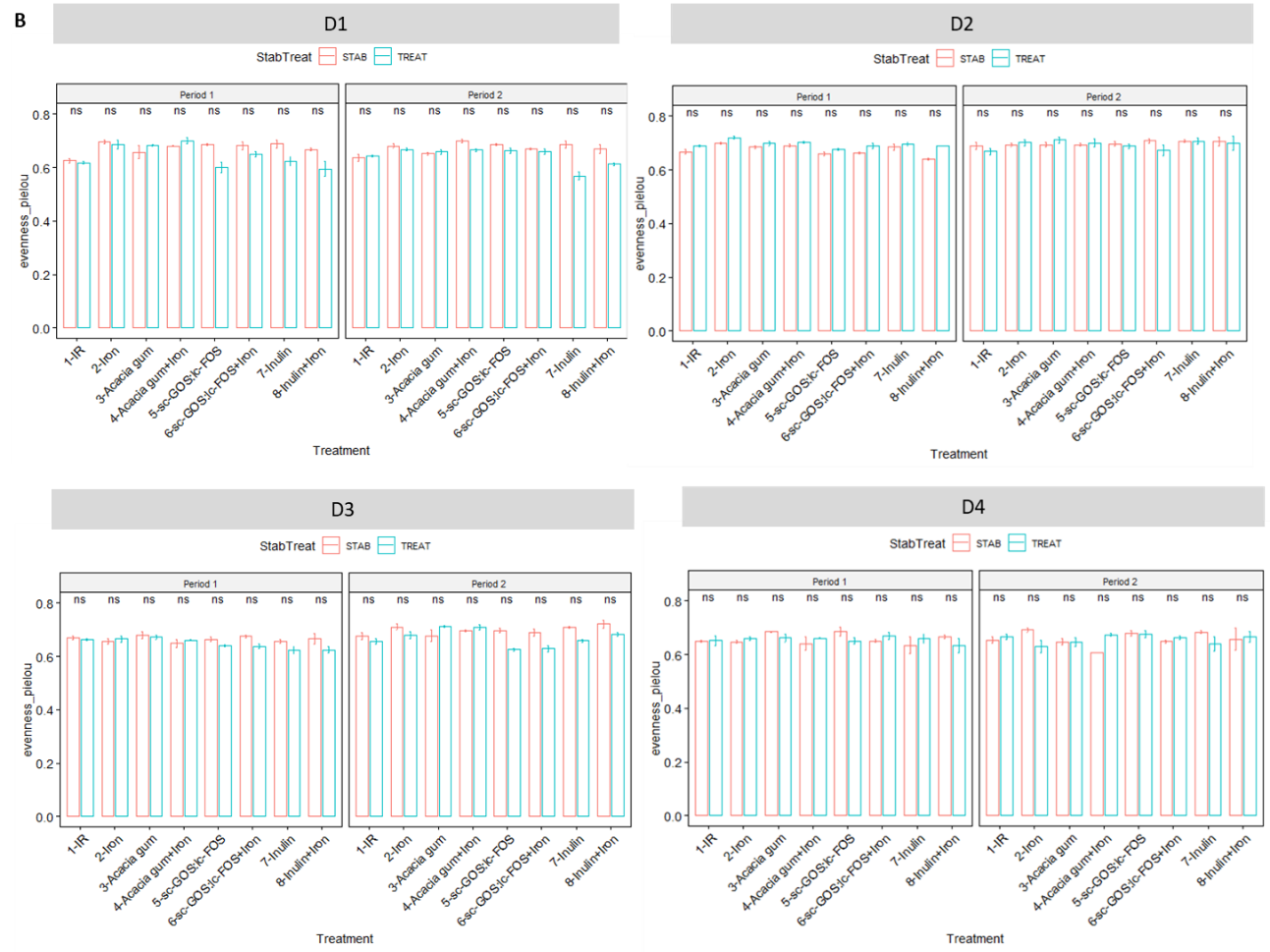
**

**Figure S4**: **Alpha diversity in stabilized and treated in vitro microbiota during prebiotic candidate screening. A** Bar plot visualizing average richness of three days assessed by observed numbers of ASV’s in all in vitro microbiota from D1, D2, D3 and D4. **B** Bar plot visualizing Pielou’s evenness index in all in vitro microbiota from D1, D2, D3 and D4. ns = no significant difference between treated and stabilized microbiota samples.


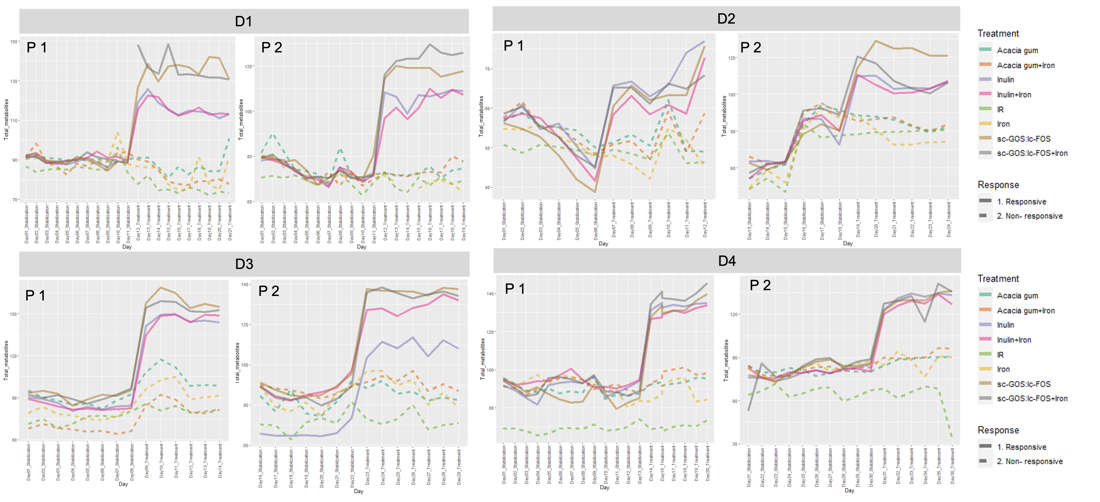


**Figure S5**: Total metabolites (mM; HPLC) over experiment course in the four in vitro donor microbiota upon stabilization and treatment during period 1 (P1, left) and period 2 (P2, right).


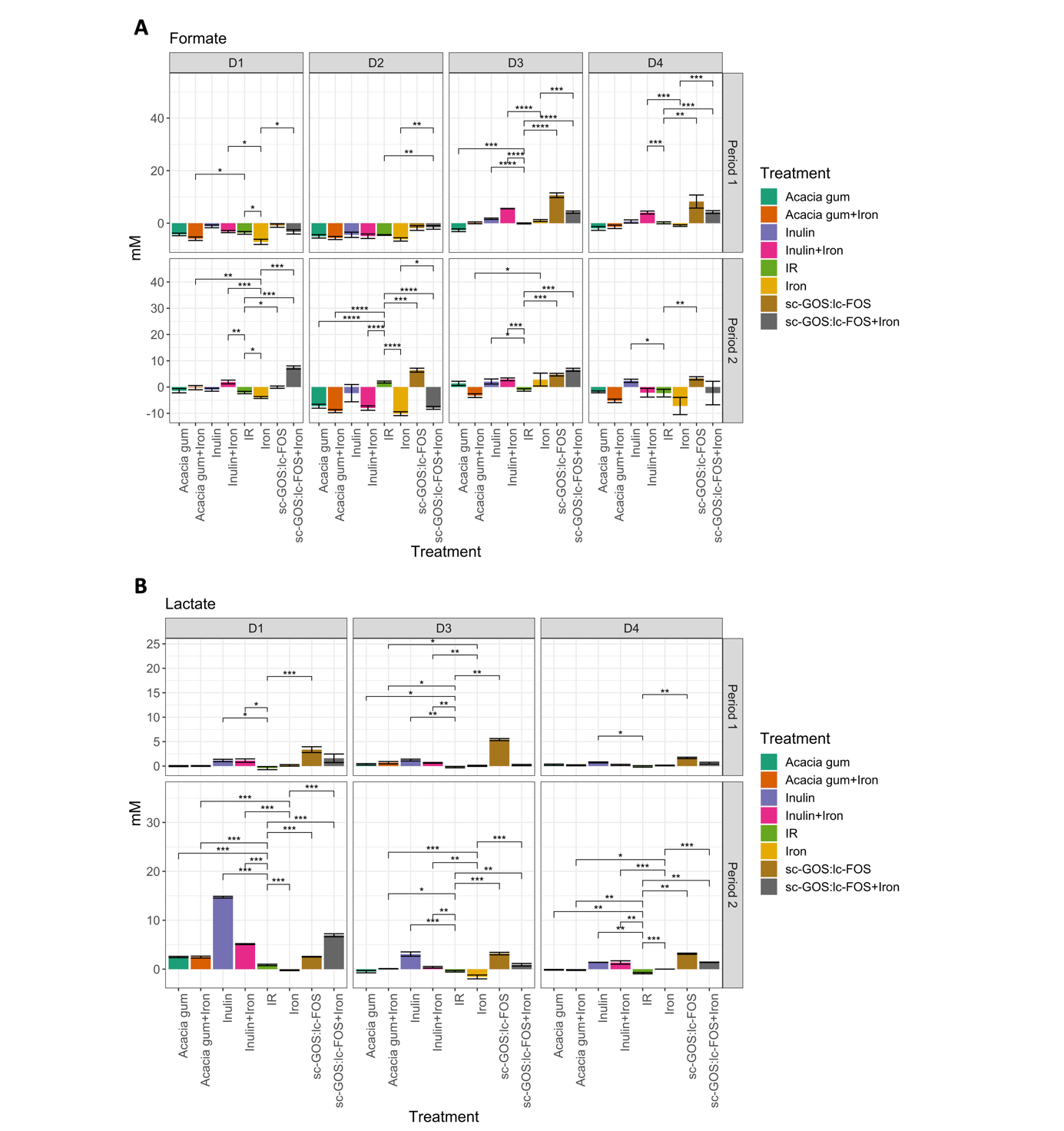


**Figure S6:** **SCFA-promoting effect of the treatments**. **A** Changes in production formate and **B** lactate (mM) upon treatments across all four donor in vitro microbiota. (Pairwise Wilcoxon rank sum test with multiple comparison correction: * p<0.05, ** p<0.01, *** p<0.005, **** p<0.001).


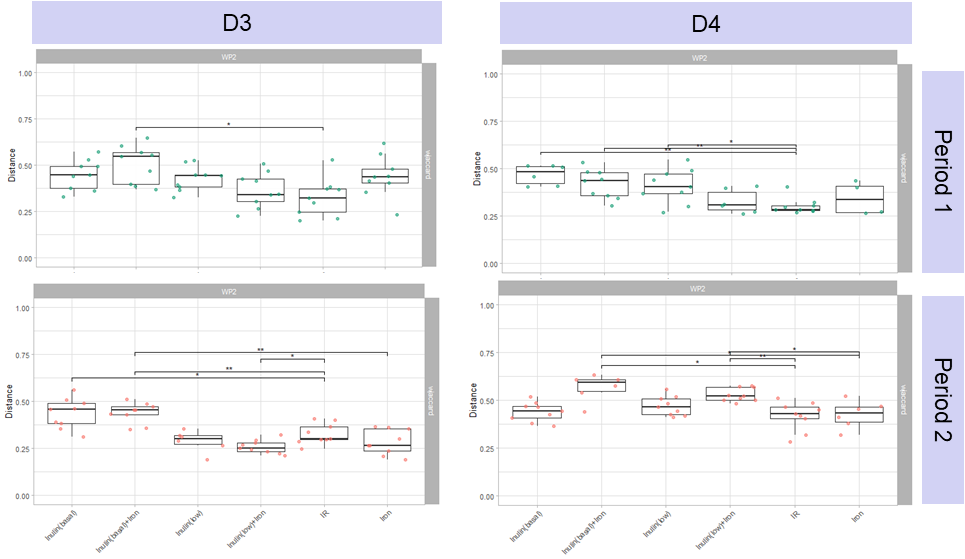


**Figure S7**: Distance box plots of weighted Jaccard distance metric assessing the treatment induced microbiota shifts for donor 3 and donor 4 in vitro microbiota in period 1 (above) and period 2 (bellow). The values indicate the distance between the community at the end of stabilization and the community at the end of the treatment within each treatment.

**Supplementary tables**

**Table S1: Metadata from Donor 1-4.**

| **Donor** | **Sex** | **Age**  **(months)** | **Birth** | **Time since weaning (months)** | **Antibiotic**  **treatment** | **Fecal pH** | **Fecal calprotectin**  **(normal: 15-50 µg/g)** |
| --- | --- | --- | --- | --- | --- | --- | --- |
| **Donor 1 (D1)** | Male | 6 | Term | 0 | No | 4.8 | 26.6 ug/g |
| **Donor 2 (D2)** | Female | 6 | Term | 0 | No | 5.32 | 251.1 ug/g |
| **Donor 3 (D3)** | Female | 8 | Term | 2.2 | No | 4.81 | 105.6 ug/g |
| **Donor 4 (D4)** | Male | 10 | Term | 4.7 | No | 7.25 | 180.7 ug/g |

**Table S2: Composition of fibers**

| **Fiber code** | **Composition** | **Purity** | **Moieties** | **Linkage** | **DP** | **Supplier** | **Content in nutritive medium** | **Concentration fiber in nutritive medium** |
| --- | --- | --- | --- | --- | --- | --- | --- | --- |
| **Acacia gum** | Acacia gum | 100% | galactose, arabinose, rhamnose, glucoronic acids | Branched, main link 1,3-linked b-D-galactopyranosyl, side link 1,6 linkage | Complex molecule, average molecular weight varying from 300 to 800 kDa | FIBREGUM LI, Nexira, France | 5.5 g/L | 5.5 g/L |
| **scGOS/lcFOS** | 90% short-chain GOS    10% long-chain FOS | 70% | glucose, galactose    fructose, glucose | b-glycosidic bonds (1-2,1-3,1-4,1-6)  b-2,1 glycosidic bonds | 2 - 10  5-60; Average DP > 24 | Vivinal GOS powder, Friesland-Campina, The Netherlands  Orafti HP, Beneo, Belgium | 7.9 g/L | 5.5 g/L |
| **Inulin/ Inulin(basal)** | Native inulin | 95% | fructose, glucose | b-2,1 glycosidic bonds | 2-60; Average DP > 10 | Orafti ST, Beneo, Belgium | 6.05 g/L | 5.5 g/L |
| **Inulin(low)** | Native inulin | 95% | fructose, glucose | b-2,1 glycosidic bonds | 2-60; Average DP > 10 | Orafti ST, Beneo, Belgium | 3.02 g/L | 2.75 g/L |

**Table S3: Primers used for PCR and qPCR analysis for fecal and fermentation samples**

| **Target** | **Primers** | **5'-3' sequence** | **Target gene** | **Amplicon length [bp]** | **Reference** |
| --- | --- | --- | --- | --- | --- |
| Total Bacteria | Eub338F | ACT CCT ACG GGA GGC AGC AG | 16S rRNA gene | 200 | (5) |
|  | Eub518R | ATT ACC GCG GCT GCT GG |  |  |  |
| *Enterobacteriaceae* | Eco1457F | CAT TGA CGT TAC CCG CAG AAG AAG C | 16S rRNA gene | 195 | (6) |
|  | Eco1652R | CTC TAC GAG ACT CAA GCT TGC |  |  |  |
| *Lactobacillus*/*Pediococcus* /*Leuconostoc* (LLP) | F_Lacto 05 | AGC AGT AGG GAA TCT TCC A | 16S rRNA gene | 375 | (7) |
|  | R_Lacto 04 | CGC CAC TGG TGT TCY TCC ATA TA |  |  |  |
| *Bifidobacterium* | Bif F | TCG CGT CYG GTG TGA AAG | 16S rRNA gene | 243 | (8) |
|  | Bif R | CCA CAT CCA GCR TCC AC |  |  |  |
| *Clostridioides difficile* | cdF | TTG AGC GAT TTA CTT CGG TAA AGA | 16S rRNA gene | 157 | (8) |
|  | cdR | CCA TCC TGT ACT GGC TCA CCT |  |  |  |
| *Clostridium perfringens* | plcF | AAG TTA CCT TTG CTG CAT AAT CCC | plc (alpha toxin) | 283 | (8) |
|  | plcR | ATA GAT ACT CCA TAT CAT CCT GCT |  |  |  |
| Enteropathogenic  *Escherichia coli* (EPEC) | EAE-a | ATG CTT AGT GCT GGT TTA GG | eaeA (E. coli attaching and effacing) | 248 | (9) |
|  | EAE-b | GCC TTC ATC ATT TCG CTT TC |  |  |  |
| *Salmonella* spp. | invA, 139 | GTG AAA TTA TCG CCA CGT TCG GGC AA | invA (invasion) | 284 | (9) |
|  | invA, 141 | TCA TCG CAC CGT CAA AGG AAC C |  |  |  |
| Enterotoxigenic  *Escherichia coli* | LT-1 | AGC AGG TTT CCC ACC GGA TCA CCA | LT (heat-labile enterotoxin) | 132 | (9) |
|  | LT-2 | GTG CTC AGA TTC TGG GTC TC |  |  |  |
| *Staphylococcus aureus* | SA-1 | GCG ATT GAT GGT GAT ACG GTT | Nuclease | 276 | (9) |
|  | SA-2 | CAA GCC TTG ACG AAC TAA AGC |  |  |  |
| Enterohemorrhagic  *Escherichia coli* | JMS1F | GTC ACA GTA ACA AAC CGT AAC A | stx 1 (shiga toxin) | 95 | (9) |
|  | JMS1R | TCG TTG ACT ACT TCT TAT CTG GA |  |  |  |
| Enterohemorrhagic  *Escherichia coli* | JMS2F | CGA CCC CTC TTG AAC ATA | stx 2 (shiga toxin) | 108 | (9) |
|  | JMS2G | GAT AGA CAT CAA GCC CTC GT |  |  |  |
| *Campylobacter* | Camp-fwd | CTG CTT AAC ACA AGT TGA GTA GG | 16S rRNA gene |  | (10) |
|  | Camp-rev | TTC CTT AGG TAC CGT CAG AA |  |  |  |

**Table S4: ASV taxonomic assignment based on National Center for Biotechnology Information (NCBI)**

| **ASV** | **Assigned taxonomy** | **Assignation confidence (%)** |
| --- | --- | --- |
| ASV0003 | *Bifidobacterium* *breve* | 100 |
|  | *Bifidobacterium* *scaligerum* | 100 |
|  | *Bifidobacterium longum subsp. Infantis* | 100 |
| ASV0008 | *Bifidobacterium* *adolescentis* | 100 |
|  | *Bifidobacterium* *breve* | 99.6 |
|  | *Bifidobacterium scaligerum* | 99.6 |
|  | *Bifidobacterium longum subsp. Infantis* | 99.6 |
| ASV0013 | *Bifidobacterium* *callitrichidarum* | 100 |
|  | *Bifidobacterium* *catenulatum* | 100 |
|  | *Bifidobacterium* *gallicum* | 100 |
|  | *Bifidobacterium* *pseudocatenulatum* | 100 |

**Table S5: Outcome from differentially abundant genera analysis with DESeq2. Significant shifts (treatment vs. stabilization) upon treatments in genera relative abundance within TRs compared to IR microbiota in period 1 and period 2.**

| **Genus** | **log2FoldChange** | **Treatment** | **Period** | **Donor** | **STAB** | **TREAT** |
| --- | --- | --- | --- | --- | --- | --- |
| *Veillonella* | -0.55335 | Inulin+Iron | Period 1 | D1 | 19.79% | 12.98% |
| *Veillonella* | -0.93664 | Inulin+Iron | Period 2 | D1 | 15.47% | 6.75% |
| *Streptococcus* | 0.559788 | sc-GOS:lc-FOS | Period 1 | D1 | 24.44% | 26.00% |
| *Streptococcus* | 0.952037 | sc-GOS:lc-FOS | Period 2 | D1 | 20.34% | 26.56% |
| *Propionibacterium* | 4.11284 | Iron | Period 1 | D1 | 0.00% | 0.06% |
| *Propionibacterium* | 5.476204 | Iron | Period 2 | D1 | 0.00% | 0.14% |
| *Propionibacterium* | 3.785475 | Acacia gum | Period 1 | D1 | 0.01% | 0.07% |
| *Propionibacterium* | 4.290108 | Acacia gum | Period 2 | D1 | 0.00% | 0.09% |
| *Propionibacterium* | 6.982136 | Acacia gum+Iron | Period 1 | D1 | 0.00% | 0.08% |
| *Propionibacterium* | 5.384246 | Acacia gum+Iron | Period 2 | D1 | 0.00% | 0.08% |
| *Propionibacterium* | 6.545716 | Inulin | Period 1 | D1 | 0.00% | 0.05% |
| *Propionibacterium* | 5.689046 | Inulin | Period 2 | D1 | 0.01% | 0.09% |
| *Morganella* | -1.41746 | Iron | Period 1 | D1 | 2.13% | 0.83% |
| *Morganella* | -1.19004 | Iron | Period 2 | D1 | 0.23% | 0.10% |
| *Morganella* | -0.85107 | Inulin | Period 1 | D1 | 1.98% | 1.07% |
| *Morganella* | -1.49971 | Inulin | Period 2 | D1 | 0.20% | 0.03% |
| *Levilactobacillus* | 5.731629 | Inulin+Iron | Period 1 | D1 | 0.00% | 0.06% |
| *Levilactobacillus* | 5.848383 | Inulin+Iron | Period 2 | D1 | 0.02% | 0.76% |
| *Enterococcus* | 3.79442 | sc-GOS:lc-FOS | Period 1 | D1 | 0.13% | 1.07% |
| *Enterococcus* | 4.654034 | sc-GOS:lc-FOS | Period 2 | D1 | 0.14% | 2.02% |
| *Enterococcus* | 4.07128 | sc-GOS:lc-FOS+Iron | Period 1 | D1 | 0.06% | 1.07% |
| *Enterococcus* | 4.757137 | sc-GOS:lc-FOS+Iron | Period 2 | D1 | 0.14% | 3.01% |
| *Enterococcus* | 1.096742 | Acacia gum+Iron | Period 1 | D1 | 0.11% | 0.18% |
| *Enterococcus* | 1.798382 | Acacia gum+Iron | Period 2 | D1 | 0.10% | 0.29% |
| *Enterococcus* | 1.451281 | Inulin | Period 1 | D1 | 0.09% | 0.16% |
| *Enterococcus* | 1.041656 | Inulin | Period 2 | D1 | 0.12% | 0.18% |
| *Enterococcus* | 1.586904 | Inulin+Iron | Period 1 | D1 | 0.06% | 0.13% |
| *Enterococcus* | 1.453445 | Inulin+Iron | Period 2 | D1 | 0.08% | 0.29% |
| *Eggerthella* | 1.55536 | sc-GOS:lc-FOS | Period 1 | D1 | 0.27% | 0.65% |
| *Eggerthella* | 2.55377 | sc-GOS:lc-FOS | Period 2 | D1 | 0.23% | 0.90% |
| *Eggerthella* | 1.76605 | sc-GOS:lc-FOS+Iron | Period 1 | D1 | 0.31% | 0.81% |
| *Eggerthella* | 1.84125 | sc-GOS:lc-FOS+Iron | Period 2 | D1 | 0.24% | 0.68% |
| *Eggerthella* | 0.984782 | Inulin | Period 1 | D1 | 0.22% | 0.42% |
| *Eggerthella* | 1.66933 | Inulin | Period 2 | D1 | 0.23% | 0.71% |
| *Eggerthella* | 0.869803 | Inulin+Iron | Period 1 | D1 | 0.34% | 0.65% |
| *Eggerthella* | 1.86682 | Inulin+Iron | Period 2 | D1 | 0.17% | 0.55% |
| *Dialister* | -0.51531 | Acacia gum | Period 1 | D1 | 1.13% | 0.75% |
| *Dialister* | -0.65398 | Acacia gum | Period 2 | D1 | 0.91% | 0.52% |
| *Clostridium sensu stricto 1* | -4.52372 | sc-GOS:lc-FOS+Iron | Period 1 | D1 | 2.81% | 0.12% |
| *Clostridium sensu stricto 1* | -0.8055 | sc-GOS:lc-FOS+Iron | Period 2 | D1 | 3.41% | 1.41% |
| *Clostridium sensu stricto 1* | -0.63863 | Inulin | Period 1 | D1 | 3.52% | 2.15% |
| *Clostridium sensu stricto 1* | -1.12843 | Inulin | Period 2 | D1 | 2.76% | 0.97% |
| *Clostridium sensu stricto 1* | -1.01588 | Inulin+Iron | Period 1 | D1 | 3.03% | 1.50% |
| *Clostridium sensu stricto 1* | -1.63144 | Inulin+Iron | Period 2 | D1 | 3.50% | 0.95% |
| *Clostridioides* | -0.88834 | Inulin | Period 1 | D1 | 2.57% | 1.24% |
| *Clostridioides* | -0.48631 | Inulin | Period 2 | D1 | 2.46% | 1.39% |
| *Bifidobacterium* | - | IR | Period 1 | D1 | 27.00% | 23.00% |
| *Bifidobacterium* | - | IR | Period 2 | D1 | 20.00% | 23.00% |
| *Bifidobacterium* | 1.383694 | sc-GOS:lc-FOS | Period 1 | D1 | 20.58% | 37.89% |
| *Bifidobacterium* | 1.236529 | sc-GOS:lc-FOS | Period 2 | D1 | 19.77% | 30.79% |
| *Bifidobacterium* | 0.515463 | sc-GOS:lc-FOS+Iron | Period 1 | D1 | 23.12% | 29.63% |
| *Bifidobacterium* | 1.235725 | sc-GOS:lc-FOS+Iron | Period 2 | D1 | 18.24% | 32.54% |
| *Bifidobacterium* | 0.568347 | Inulin | Period 1 | D1 | 22.80% | 32.16% |
| *Bifidobacterium* | 1.120023 | Inulin | Period 2 | D1 | 19.79% | 33.47% |
| *Bifidobacterium* | 0.403528 | Inulin+Iron | Period 1 | D1 | 26.72% | 34.33% |
| *Bifidobacterium* | 1.117583 | Inulin+Iron | Period 2 | D1 | 18.66% | 34.11% |
| *Peptoniphilus* | -0.585723124 | Iron | Period 1 | D2 | 1.03% | 0.60% |
| *Peptoniphilus* | -2.537035745 | Iron | Period 2 | D2 | 1.05% | 0.18% |
| *Peptoniphilus* | -0.701918656 | sc-GOS:lc-FOS+Iron | Period 1 | D2 | 0.92% | 0.38% |
| *Peptoniphilus* | -0.872792147 | sc-GOS:lc-FOS+Iron | Period 2 | D2 | 0.72% | 0.30% |
| *Peptoniphilus* | -0.999789682 | Acacia gum+Iron | Period 1 | D2 | 1.13% | 0.55% |
| *Peptoniphilus* | -1.657164676 | Acacia gum+Iron | Period 2 | D2 | 1.00% | 0.34% |
| *Peptoniphilus* | -1.131956319 | Acacia gum | Period 1 | D2 | 0.90% | 0.42% |
| *Peptoniphilus* | -1.452388488 | Acacia gum | Period 2 | D2 | 0.96% | 0.37% |
| *Peptoniphilus* | -2.16660536 | Inulin+Iron | Period 1 | D2 | 0.90% | 0.20% |
| *Peptoniphilus* | -1.500093714 | Inulin+Iron | Period 2 | D2 | 0.74% | 0.25% |
| *Olsenella* | -0.678108196 | sc-GOS:lc-FOS | Period 1 | D2 | 9.21% | 4.36% |
| *Olsenella* | -0.598071387 | sc-GOS:lc-FOS | Period 2 | D2 | 7.43% | 4.35% |
| *Megasphaera* | 1.791816905 | sc-GOS:lc-FOS | Period 1 | D2 | 5.85% | 15.07% |
| *Megasphaera* | 0.94917847 | sc-GOS:lc-FOS | Period 2 | D2 | 8.98% | 15.48% |
| *Hungatella* | -0.760219668 | sc-GOS:lc-FOS | Period 1 | D2 | 7.04% | 3.07% |
| *Hungatella* | -0.760219668 | sc-GOS:lc-FOS | Period 2 | D2 | 4.06% | 2.68% |
| *Hungatella* | -1.500454067 | Inulin+Iron | Period 1 | D2 | 6.89% | 2.46% |
| *Hungatella* | -0.912503421 | Inulin+Iron | Period 2 | D2 | 4.29% | 2.19% |
| *Hungatella* | -1.394247627 | Inulin | Period 1 | D2 | 5.86% | 2.18% |
| *Hungatella* | -0.80626624 | Inulin | Period 2 | D2 | 4.04% | 2.28% |
| *Flavonifractor* | -0.398873626 | sc-GOS:lc-FOS | Period 1 | D2 | 2.90% | 1.69% |
| *Flavonifractor* | -0.441193073 | sc-GOS:lc-FOS | Period 2 | D2 | 2.99% | 1.90% |
| *Flavonifractor* | -0.377195527 | Acacia gum+Iron | Period 1 | D2 | 3.37% | 2.56% |
| *Flavonifractor* | -0.403851283 | Acacia gum+Iron | Period 2 | D2 | 3.16% | 2.45% |
| *Eubacterium* | 0.911499752 | Acacia gum+Iron | Period 1 | D2 | 0.13% | 0.19% |
| *Eubacterium* | 0.788569057 | Acacia gum+Iron | Period 2 | D2 | 0.12% | 0.20% |
| *Escherichia-Shigella* | -3.474285883 | IR | Period 1 | D2 | 0.06% | 0.00% |
| *Escherichia-Shigella* | -3.139125109 | IR | Period 2 | D2 | 0.12% | 0.01% |
| *Dialister* | 0.85764884 | sc-GOS:lc-FOS+Iron | Period 1 | D2 | 1.66% | 2.30% |
| *Dialister* | 0.944459144 | sc-GOS:lc-FOS+Iron | Period 2 | D2 | 1.76% | 2.72% |
| *Clostridioides* | 4.532178644 | Iron | Period 1 | D2 | 0.03% | 0.57% |
| *Clostridioides* | 2.628171966 | Iron | Period 2 | D2 | 0.01% | 0.08% |
| *Clostridioides* | 1.248070962 | Acacia gum+Iron | Period 1 | D2 | 0.03% | 0.08% |
| *Clostridioides* | 1.627913676 | Acacia gum+Iron | Period 2 | D2 | 0.03% | 0.08% |
| *Bifidobacterium* | 3.198388967 | sc-GOS:lc-FOS+Iron | Period 1 | D2 | 0.66% | 4.67% |
| *Bifidobacterium* | 1.777809826 | sc-GOS:lc-FOS+Iron | Period 2 | D2 | 2.38% | 6.46% |
| *Bifidobacterium* | 3.935172506 | sc-GOS:lc-FOS | Period 1 | D2 | 0.68% | 8.09% |
| *Bifidobacterium* | 1.552863794 | sc-GOS:lc-FOS | Period 2 | D2 | 2.52% | 6.67% |
| *Bifidobacterium* | 3.376993939 | Inulin+Iron | Period 1 | D2 | 0.83% | 8.32% |
| *Bifidobacterium* | 1.98865979 | Inulin+Iron | Period 2 | D2 | 2.28% | 9.04% |
| *Lactococcus* | 5.286750637 | Inulin | Period 1 | D3 | 0.53% | 15.71% |
| *Lactococcus* | 1.749408426 | Inulin | Period 2 | D3 | 5.86% | 13.98% |
| *Flavonifractor* | 0.696552881 | sc-GOS:lc-FOS | Period 1 | D3 | 5.59% | 7.36% |
| *Flavonifractor* | 0.674078446 | sc-GOS:lc-FOS | Period 2 | D3 | 4.63% | 6.35% |
| *Eubacterium* | 7.475338127 | sc-GOS:lc-FOS+Iron | Period 1 | D3 | 0.00% | 0.43% |
| *Eubacterium* | 1.655944325 | sc-GOS:lc-FOS+Iron | Period 2 | D3 | 0.78% | 1.72% |
| *Eubacterium* | 7.636253712 | sc-GOS:lc-FOS | Period 1 | D3 | 0.00% | 0.40% |
| *Eubacterium* | 1.147731791 | sc-GOS:lc-FOS | Period 2 | D3 | 0.53% | 0.89% |
| *Eubacterium* | 8.084067067 | Acacia gum+Iron | Period 1 | D3 | 0.00% | 0.59% |
| *Eubacterium* | 1.182402688 | Acacia gum+Iron | Period 2 | D3 | 0.93% | 1.83% |
| *Eubacterium* | 5.403337 | Acacia gum | Period 1 | D3 | 0.01% | 0.58% |
| *Eubacterium* | 1.556996661 | Acacia gum | Period 2 | D3 | 0.35% | 1.53% |
| *Enterococcus* | - | Iron | Period 1 | D3 | 3.34% | 0.65% |
| *Enterococcus* | - | Iron | Period 2 | D3 | 1.76% | 0.28% |
| *Enterococcus* | -2.435827626 | IR | Period 1 | D3 | 1.28% | 0.19% |
| *Enterococcus* | -2.861145016 | IR | Period 2 | D3 | 1.03% | 0.12% |
| *Enterococcus* | -2.379371246 | sc-GOS:lc-FOS+Iron | Period 1 | D3 | 4.21% | 0.78% |
| *Enterococcus* | -2.960437008 | sc-GOS:lc-FOS+Iron | Period 2 | D3 | 1.90% | 0.26% |
| *Enterococcus* | -2.895151533 | Acacia gum+Iron | Period 1 | D3 | 1.48% | 0.11% |
| *Enterococcus* | -3.16460225 | Acacia gum+Iron | Period 2 | D3 | 1.43% | 0.18% |
| *Enterococcus* | -2.888070397 | Acacia gum | Period 1 | D3 | 2.03% | 0.25% |
| *Enterococcus* | -2.136032824 | Acacia gum | Period 2 | D3 | 0.90% | 0.23% |
| *Collinsella* | 0.811501649 | sc-GOS:lc-FOS | Period 1 | D3 | 14.40% | 20.66% |
| *Collinsella* | 0.785117832 | sc-GOS:lc-FOS | Period 2 | D3 | 18.09% | 28.70% |
| *Bifidobacterium* | 1.801037381 | sc-GOS:lc-FOS | Period 1 | D3 | 6.53% | 17.22% |
| *Bifidobacterium* | 1.568535863 | sc-GOS:lc-FOS | Period 2 | D3 | 5.83% | 15.39% |
| *Bifidobacterium* | 2.364340654 | Inulin+Iron | Period 1 | D3 | 6.29% | 26.26% |
| *Bifidobacterium* | 1.473163728 | Inulin+Iron | Period 2 | D3 | 5.93% | 22.54% |
| *Bifidobacterium* | 2.109198854 | Inulin | Period 1 | D3 | 7.32% | 22.68% |
| *Bifidobacterium* | 2.669983942 | Inulin | Period 2 | D3 | 4.45% | 21.41% |
| *Bacteroides* | -0.444208501 | sc-GOS:lc-FOS | Period 1 | D3 | 21.63% | 13.01% |
| *Bacteroides* | -0.655079063 | sc-GOS:lc-FOS | Period 2 | D3 | 17.08% | 9.50% |
| *Bacteroides* | -1.776007262 | Inulin+Iron | Period 1 | D3 | 20.89% | 4.82% |
| *Bacteroides* | -1.661782183 | Inulin+Iron | Period 2 | D3 | 18.78% | 6.96% |
| *Bacteroides* | -1.223443085 | Inulin | Period 1 | D3 | 23.41% | 7.70% |
| *Bacteroides* | -1.616413179 | Inulin | Period 2 | D3 | 16.58% | 4.10% |

**References**

1. Stoddard SF, Smith BJ, Hein R, Roller BRK, Schmidt TM. rrnDB: Improved tools for interpreting rRNA gene abundance in bacteria and archaea and a new foundation for future development. Nucleic Acids Res. 2015;43(D1):D593–8.

2. Isenring J, Geirnaert A, Hall AR, Jans C, Lacroix C, Stevens MJA. In Vitro Gut Modeling as a Tool for Adaptive Evolutionary Engineering of Lactiplantibacillus plantarum . mSystems. 2021;6(2).

3. Pham VT, Chassard C, Rifa E, Braegger C, Geirnaert A, Rocha Martin VN, et al. Lactate Metabolism Is Strongly Modulated by Fecal Inoculum, pH, and Retention Time in PolyFermS Continuous Colonic Fermentation Models Mimicking Young Infant Proximal Colon. mSystems. 2019 May 28;4(4).

4. McMurdie PJ, Holmes S. Phyloseq: An R Package for Reproducible Interactive Analysis and Graphics of Microbiome Census Data. PLoS One. 2013;8(4).

5. Fierer N, Jackson JA, Vilgalys R, Jackson RB. Assessment of soil microbial community structure by use of taxon-specific quantitative PCR assays. Appl Environ Microbiol. 2005 Jul;71(7):4117–20.

6. Bartosch S, Fite A, Macfarlane GT, McMurdo MET. Characterization of bacterial communities in feces from healthy elderly volunteers and hospitalized elderly patients by using real-time PCR and effects of antibiotic treatment on the fecal microbiota. Appl Environ Microbiol. 2004 Jun;70(6):3575–81.

7. Furet JP, Firmesse O, Gourmelon M, Bridonneau C, Tap J, Mondot S, et al. Comparative assessment of human and farm animal faecal microbiota using real-time quantitative PCR. FEMS Microbiol Ecol. 2009 Jun;68(3):351–62.

8. Rinttilä T, Kassinen A, Malinen E, Krogius L, Palva A. Development of an extensive set of 16S rDNA-targeted primers for quantification of pathogenic and indigenous bacteria in faecal samples by real-time PCR. J Appl Microbiol. 2004;97(6):1166–77.

9. Fukushima H, Tsunomori Y, Seki R. Duplex real-time SYBR green PCR assays for detection of 17 species of food- or waterborne pathogens in stools. J Clin Microbiol. 2003 Nov;41(11):5134–46.

10. Josefsen MH, Jacobsen NR, Hoorfar J. Enrichment followed by quantitative PCR both for rapid detection and as a tool for quantitative risk assessment of food-borne thermotolerant campylobacters. Appl Environ Microbiol. 2004 Jun;70(6):3588–92.
